# Low mutation rate of spontaneous mutants enables detection of causative genes by comparing whole genome sequences

**DOI:** 10.3389/fpls.2024.1366413

**Published:** 2024-04-04

**Authors:** Mao Suganami, Soichi Kojima, Hideki Yoshida, Masaki Mori, Mayuko Kawamura, Eriko Koketsu, Makoto Matsuoka

**Affiliations:** ^1^ Faculty of Food and Agricultural Sciences, Institute of Fermentation Sciences, Fukushima University, Fukushima, Japan; ^2^ Graduate School of Agricultural Science, Tohoku University, Sendai, Japan; ^3^ Bioscience and Biotechnology Center, Nagoya University, Nagoya, Japan

**Keywords:** awn, mutation rate, rice, spontaneous mutation, whole genome sequences, in silico gene isolation, gene bank

## Abstract

In the early 1900s, mutation breeding to select varieties with desirable traits using spontaneous mutation was actively conducted around the world, including Japan. In rice, the number of fixed mutations per generation was estimated to be 1.38-2.25. Although this low mutation rate was a major problem for breeding in those days, in the modern era with the development of next-generation sequencing (NGS) technology, it was conversely considered to be an advantage for efficient gene identification. In this paper, we proposed an *in silico* approach using NGS to compare the whole genome sequence of a spontaneous mutant with that of a closely related strain with a nearly identical genome, to find polymorphisms that differ between them, and to identify the causal gene by predicting the functional variation of the gene caused by the polymorphism. Using this approach, we found four causal genes for the dwarf mutation, the round shape grain mutation and the awnless mutation. Three of these genes were the same as those previously reported, but one was a novel gene involved in awn formation. The novel gene was isolated from Bozu-Aikoku, a mutant of Aikoku with the awnless trait, in which nine polymorphisms were predicted to alter gene function by their whole-genome comparison. Based on the information on gene function and tissue-specific expression patterns of these candidate genes, Os03g0115700/LOC_Os03g02460, annotated as a short-chain dehydrogenase/reductase SDR family protein, is most likely to be involved in the awnless mutation. Indeed, complementation tests by transformation showed that it is involved in awn formation. Thus, this method is an effective way to accelerate genome breeding of various crop species by enabling the identification of useful genes that can be used for crop breeding with minimal effort for NGS analysis.

## 1 Introduction

The rediscovery of Mendel’s laws of heredity in 1900, followed by the establishment of Johansen’s “pure lineage theory” in 1903, marked the beginning of modern plant breeding, in which mutation breeding was attempted in various crops. While mutation breeding with treatments that increase mutation rates, such as gamma rays, heavy ion beams, and ethyl methanesulfonate (EMS) (Jankowicz-Cieslak et al., 2016), has become common in the modern era, mutation breeding at that time depended on spontaneous mutation under natural conditions. Although the extremely low frequency of spontaneous mutation was a major problem for mutation breeding, public experimental institutions in various countries, including Japan, actively tried to find mutant lines with desirable traits and isolate pure lines from them, and several lines have been preserved to the present day.

With the recent remarkable development of next-generation sequencing (NGS) technology, it has become possible to obtain whole-genome information of the various rice lines at relatively low cost, enabling genetic and molecular biological analysis (Guo et al., 2014; Nguyen et al., 2019; Kalendar et al., 2022). In such a situation, the low frequency of spontaneous mutation could be an advantage in finding causative genes involved in traits that differ from the parental varieties. Based on this assumption, we propose an *in silico* approach to identify the causative gene by comparing the whole genome sequence of a spontaneous mutant line with that of closely related varieties, with almost identical genomes. In this study, using several spontaneous mutant lines that have been isolated in Japan, we succeeded in identifying four causative genes, including a novel gene involved in awn formation. Thus, this method offers the possibility of finding genes involved in traits that breeders are looking for with virtually no effort other than NGS analysis, and can be applied not only for rice but also for other crops.

## 2 Materials and methods

### 2.1 Plant materials and genotyping

We obtained rice varieties used in this study from NARO genebank (https://www.gene.affrc.go.jp/databases-plant_search_char.php?type=428). DNA preparation and genotyping were performed as previously described (Yano et al., 2016; Suganami et al., 2023) with slight modifications. DNA for genotyping was isolated from leaves using a DNeasy Plant Mini Kit (Qiagen, #69104) and fragmented into approximately 500 bp using Covaris S2 (Covaris). The NEBNext DNA Library Prep Reagent Set (BioLabs, #E6000) was used for DNA library construction. Paired-end sequencing was performed using the Illumina Hiseq system (Illumina Co., Ltd) with a read length of 100–150 bp. All reads were mapped against Os-NipponbareReference-IRGSP-1.0 pseudomolecules (all.con ver.7, Kawahara et al., 2013), and fastq files were converted into samfiles using the bwa-mem command of BWA software ver 0.7.18 (Li, 2013). Commands samtools-view, samtools-sort, and samtools-index of Samtools software ver1.9 (Li et al., 2009) were used to generate, sort, and index bam files successively. The variants for each accession were called using the GATK HaplotypeCaller (release 4.1.9.0) with the ‘.g.vcf’ extension (McKenna et al., 2010). GATK CombineGVCFs was used for joint genotyping to produce a single VCF file for each compared pairs and groups. Homozygous polymorphisms in all compared genomes were used for prediction of causative polymorphisms.

### 2.2 Phenotypic data

Data on culm length and ratio of length to width in brown rice were obtained from the Genebank Project, NARO (2023). Phenotypic data were scored according to an in-house manual (available from the NARO Genebank website), and the average of five replicates was recorded as a single data. We treated the lines with the same name but different JP number (i.e., index number in NARO) as different lines. For the lines with multiple data, all data are shown.

### 2.3 Transgenic analysis

Transgenic analysis was performed according to Yano et al. (2016) and Yoshida et al. (2022). To produce the complementation construct, the genomic DNA fragments of Os06g0695900/LOC_Os06g48065 and Os03g0115700/LOC_Os03g02460 plus upstream and downstream regions were PCR amplified from genomic DNA extracted from Aikoku genomic DNA. Os06g0695900/LOC_Os06g48065 genomic fragment was produced with the primer pair 5’- CGGCGCGCCGAATTCATGACATATTCTAGTACGAT-3’ and 5’- GCAGGTCGACGGATCCACACGCATACGACCAGCT-3’. Os03g0115700/LOC_Os03g02460 genomic fragment was produced with the primer pair 5’- CGGCGCGCCGAATTCAATTAGGAACTTAGGATATG-3’ and 5’- GCAGGTCGACGGATCCTGTACCTCCTTGGATGGAA-3’. The coding sequences of these genes were produced by PCR using Aikoku cDNA as template. Os06g0695900/LOC_Os06g48065 cDNA fragment was produced with the primer pair 5’- CTAGACCCGGGGATCCATGGAGCCGTCGCGGCGG-3’ and 5’- TAGCGTTAACACTAGTCTAGGTGCTAGGGCCGTT-3’. Os03g0115700/LOC_Os03g02460 cDNA fragment was produced with the primer pair 5’- CTAGACCCGGGGATCCATGCTGCGGGCGGCGAAG-3’ and 5’- TAGCGTTAACACTAGTTCAGGGAGCGGAGGCATC-3’. The genomic and CDS fragments were subcloned into the EcoRI-BamHI sites of pUbi-omega/pCAMBIA (Hirano et al., 2017) and BamHI-SpeI site of pCAMBIA1380 using the NEBuilder HiFi DNA Assembly master mix (New England Biolabs), respectively. PCR-amplified fragments were sequenced to ensure that no mutations were introduced.

### 2.4 Calculation of genomic similarity rate

The genomic similarity was calculated by dividing the number of positions where the two accessions have different alleles by the length of the reference rice genome. The number of allelic positions was counted from VCF files containing the allele information for the two accessions.

### 2.5 Prediction of causative polymorphisms

A list of polymorphisms specific to the target mutant line was prepared as the VCF file, and the effect of the polymorphisms on gene function was evaluated by SnpEff (Cingolani et al., 2012) to narrow down the list to those polymorphisms that were evaluated as HIGH or MODERATE. For the listed genes, causative polymorphisms were predicted based on gene expression information from RiceXPro (Sato et al., 2013; https://ricexpro.dna.affrc.go.jp/) and Transcriptome ENcyclopedia Of Rice (Kawahara et al., 2016; https://tenor.dna.affrc.go.jp/), gene function information from RAP-DB (https://rapdb.dna.affrc.go.jp/), Rice Genome Annotation Project (http://rice.uga.edu/), Ensembl Plants (https://plants.ensembl.org/) and Phytozome (phytozome-next.jgi.doe.gov) and the literatures, and amino acid alignment analysis using land plants as comparative targets. For amino acid alignment of sequences, Clustal Omega was used with default parameter settings (Sievers et al., 2011). The following land plant species were chosen for the alignment analysis: *Arabidopsis thaliana*, *Glycine max*, *Solanum lycopersicum L.*, *Zea mays*, *Sorghum bicolor*, *Setaria italica*, *Paspalum virgatum*, *Selaginella moellendorffii*, and *Physcomitrium patens*.

## 3 Result

**Figure 1:**
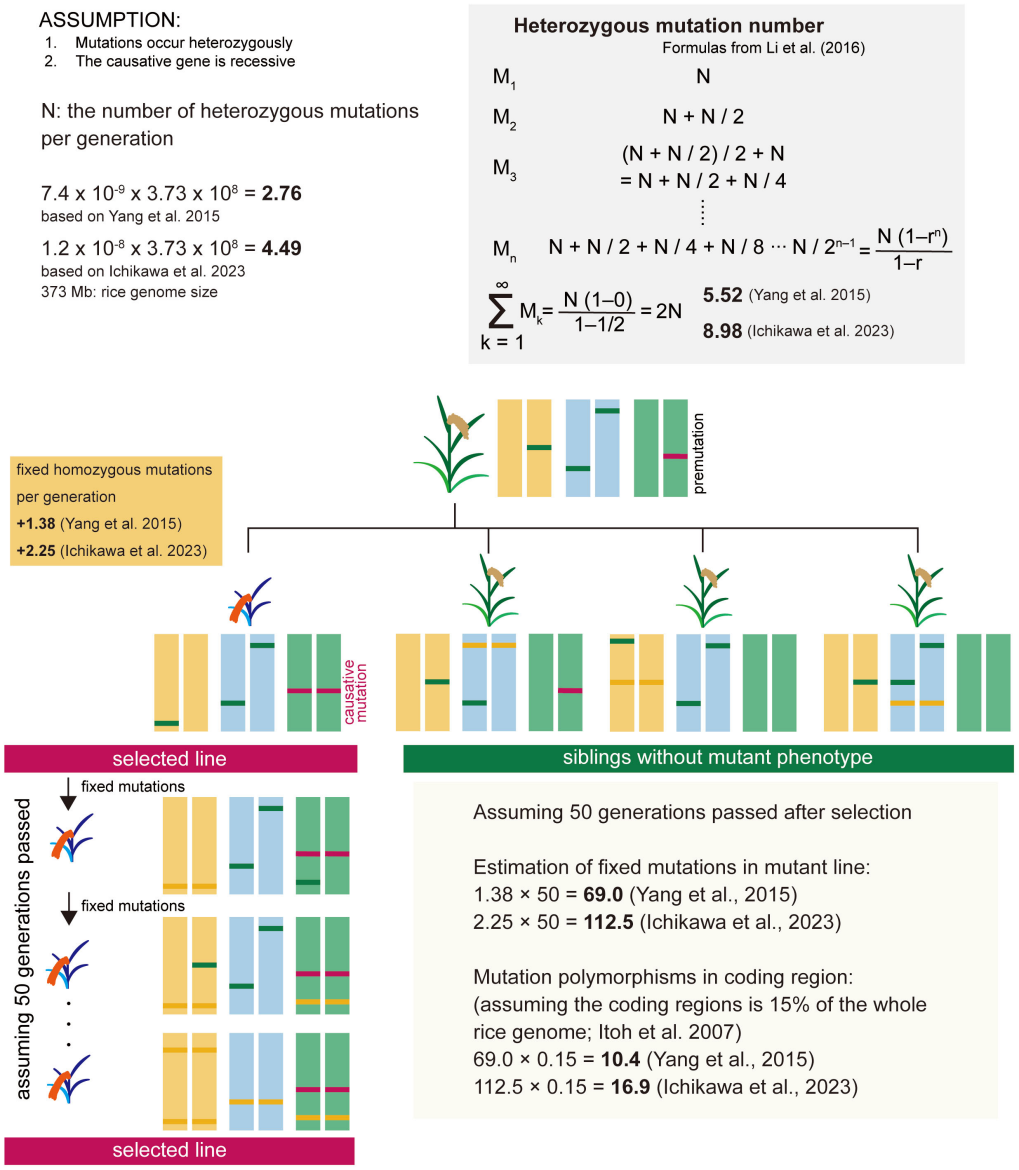
Estimation of the number of fixed spontaneous mutations in the rice mutant line. For estimating the number of fixed spontaneous mutations, we assume that mutations occur heterozygously and that the causative gene is recessive. The causative mutation in the mutant line is derived from heterozygous mutations present in the premutation line. The fixed homozygous mutations per generation are estimated to be one-quarter of the heterozygous mutations in the previous generation, i.e., N/2. Based on the reports of spontaneous mutation rate in rice (Yang et al., 2015; Ichikawa et al., 2023), the number of fixed mutations per generation is estimated to be 1.38-2.25, and when 50 generations have passed, the mutations are estimated 69.0-112.5 in the whole genome, and 10.4-16.9 in the coding region.

First, we estimated the number of mutations that occur within 373 Mb of the rice entire genome per generation (**Figure 1**). For estimating the number of fixed spontaneous mutations, we assume that mutations occur heterozygously and that the causative gene is recessive. The number of heterozygous mutations that newly occur per generation (N) is the spontaneous mutation rate x genome size. Heterozygous mutations are the sum of newly occurring mutations and mutations inherited from heterozygous mutations in the previous generation (Li et al., 2016). As shown in the gray shaded area of **Figure 1**, the number of heterozygous mutations can be expressed as the sum of a geometric progression with an initial value of N and a tolerance (r) of 1/2. Spontaneous mutation is a constantly occurring phenomenon, and heterozygous mutation should be considered to have reached saturation in an individual plant. Thus, the number of heterozygous mutations per individual plant is estimated to be 2N. The causative mutation in the mutant line is derived from heterozygous mutations present in the premutation line. The fixed homozygous mutations per generation are estimated to be one-quarter of the heterozygous mutations in the previous generation, i.e., N/2, according to Mendel’s laws. Based on the reports of spontaneous mutation rate in rice (Yang et al., 2015; Ichikawa et al., 2023), the number of fixed mutations per generation is estimated to be 1.38-2.25, and when 50 generations have passed, the number of mutations is estimated to be 69.0-112.5 in the whole genome, and 10.4-16.9 in the coding region based on Itoh et al. (2007) (**Figure 1**). Furthermore, by eliminating mutations that are not involved in changes in gene function, including non-synonymous substitutions, the number of candidate causative polymorphisms can be reduced to ~10. Based on this speculation, we calculated that approximately 10 to 17 genes would be mutated, and by examining the surrounding information on the effects of these mutations on gene function and tissue-specific expression patterns, etc., we thought it highly likely that we would be able to identify the causative gene.

**Figure 2:**
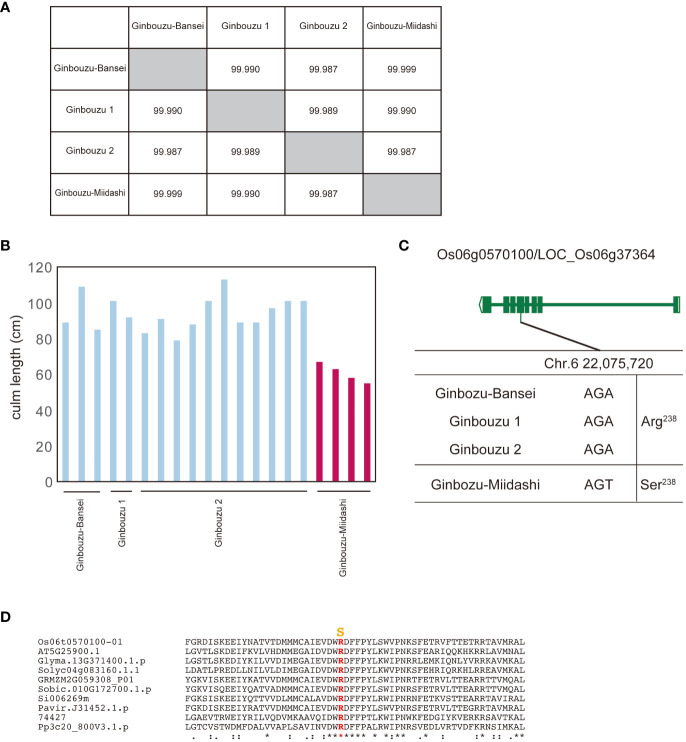
Identification of genes for dwarf phenotype from Ginbouzu varieties by comparison of their whole genomes. **(A)** Genomic similarity among three nondwarf cultivars (Ginbouzu-Bansei, Ginbouzu 1 and Ginbouzu 2) and dwarf cultivar, Ginbouzu-Miidashi. **(B)** Culm length in three nondwarf cultivars and Ginbouzu-Miidashi. **(C)** Exon-intron structure of Os06g0570100/LOC_Os06g37364 with DNA polymorphisms including amino acid exchanges. ID-1, DKT-1,DKT-2, DK22, and CM 1361-1 are mutations reported in Fujisawa et al. (1999). **(D)** Amino acid alignment of Os06g0570100 surrounding mutation site. Mutations shown in yellow are thought to be the causative amino acid substitution for culm length. Os06t0570100-01 (*Oryza sativa*), AT5G25900.1 (*Arabidopsis thaliana*), Glyma.13G371400.1.p (*Glycine max*), Solyc04g083160.1.1 (*Solanum lycopersicum L.*), GRMZM2G059308_P01 (*Zea mays*), Sobic.010G172700.1.p (*Sorghum bicolor*), Si006269m (*Setaria italica*), Pavir.J31452.1.p (*Paspalum virgatum*), 74427 (*Selaginella moellendorffii*), Pp3c20_800V3.1.p (*Physcomitrium patens*). The overall amino acid alignment of Os06g0570100 is shown in **Supplementary Figure 1**. They mean the similarity of amino acid sequences. * means identical, : means almost identical

For this study, we used data from NARO genebank (https://www.gene.affrc.go.jp/databases-plant_search_char.php?type=428), which has registered varieties selected through a mutation selection process from previous breeding programs in Japan. Our first study focused on Ginbouzu-Miidashi, which was isolated in 1919 as a dwarf mutant of Ginbouzu (Terada, 1955). Its entire genome is almost identical to the other three Ginbouzu varieties (**Figure 2A**), but Ginbouzu-Miidashi has a shorter culm length compared to the others (**Figure 2B**), as shown in the old literature. By comparing the whole genome sequence between Ginbouzu-Miidashi and the other Ginbouzu varieties, we identified polymorphisms specific to Ginbouzu-Miidashi and scored these as HIGH (frameshift, stop codon gain/lost) or MODERATE (amino acid substitution, inframe insertion/deletion) by snpEff (Cingolani et al., 2012). As a result, 22 polymorphisms in nine genes were identified as candidate causative polymorphisms (**Supplementary Table 1**). Based on information on gene function, gene expression, and the alignment of amino acid substitution (details shown in **Supplementary Table 1**), the amino acid substitution R238S in Os06g0570100/LOC_Os06g37364 was determined to be the causal polymorphism (**Figure 2C**). The residue R238 is highly conserved in land plants (**Figure 2D**, **Supplementary Figure 1**). As this polymorphism has already been reported as the cause of dwarfism in Tan-Ginbouzu (D35/KO2; Itoh et al., 2004), this supports the validity of this approach.

**Figure 3:**
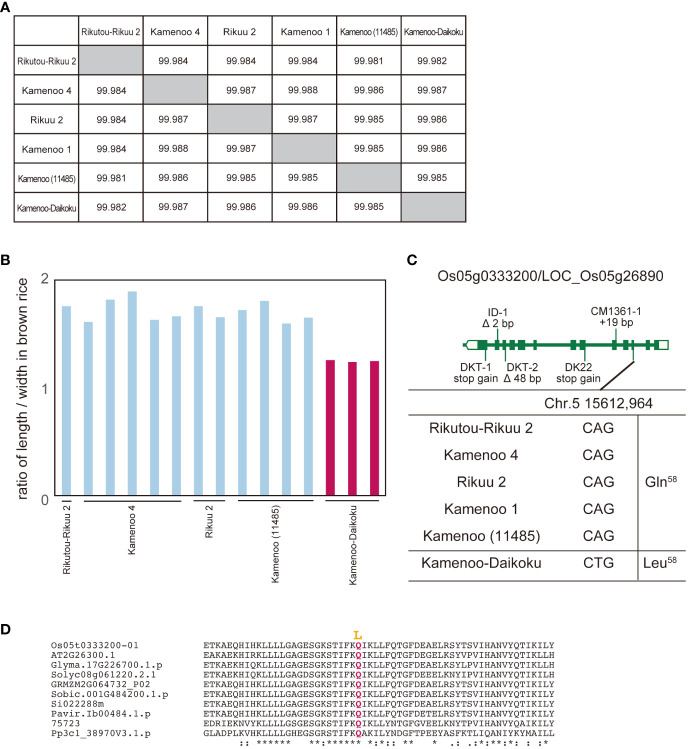
Identification of genes for Daikoku (short grain) phenotype from Kamenoo varieties by comparison of their whole genomes. **(A)** Genomic similarity rate among five normal grain cultivars (Rikutou-Rikuu 2, Kamenoo 4, Rikuu 2, Kamenoo 1, Kamenoo (11485)) and Daikoku variety, Kamenoo-Daikoku. **(B)** Ratio of length to width in brown rice in four normal grain cultivars and Kamenoo-Daikoku. **(C)** Exon-intron structure of Os05g0333200/LOC_Os05g26890 with DNA polymorphisms including amino acid substitution. **(D)** Amino acid alignment of Os05g0333200 surrounding mutation site. Mutations shown in yellow are thought to be the causative amino acid substitution for short grain. Os05t0333200-01 (*Oryza sativa*), AT2G26300.1 (*Arabidopsis thaliana*), Glyma.17G226700.1.p (*Glycine max*), Solyc08g061220.2.1 (*Solanum lycopersicum L.*), GRMZM2G064732_P02 (*Zea mays*), Sobic.001G484200.1.p (*Sorghum bicolor*), Si022288m (*Setaria italica*), Pavir.Ib00484.1.p (*Paspalum virgatum*), 75723 (*Selaginella moellendorffii*), Pp3cl_38907V3.1.p (*Physcomitrium patens*). The overall amino acid alignment of Os05g0333200 is shown in **Supplementary Figure 2**. They mean the similarity of amino acid sequences. * means identical, : means almost identical

In a second study, we focused on Kamenoo-Daikoku. According to its name, we assumed that Kamenoo-Daikoku was selected for the “Daikoku (short grain in Japanese)” phenotype (Hayashi and Takamure, 1999). We compared the whole genome sequence and grain length between Kamenoo-Daikoku and five Kamenoo and related varieties, including Rikutou-Rikuu 2 and Rikuu 2 (almost identical genome to Kamenoo) (**Figures 2A, B**). Seventeen polymorphisms in eight genes were identified as candidate causative polymorphisms by the above prediction method (**Supplementary Table 2**). By comprehensive evaluation, the amino acid substitution Q58L in Os05g0333200/LOC_Os05g26890 was judged to be the causative polymorphism (**Figure 3C**). Because the residue Q58 is highly conserved in land plants, this amino acid substitution was predicted to be deleterious (**Figure 2D**, **Supplementary Figure 2**). This gene has been reported as the causative gene for five different mutants showing the same short grain phenotypes (Fujisawa et al., 1999). The five mutants analyzed in this paper by Fujisawa et al. (1999) all had different mutations occurring independently in the same gene, but none of them contained the Kamenoo-Daikoku mutation found here. The short-grain rice produced by the Daikoku mutation is not considered to be an agriculturally useful mutation because of its smaller grain size and lower yield, but if the mutant lines are maintained, the gene responsible for it can be easily identified by the method we propose.

**Figure 4:**
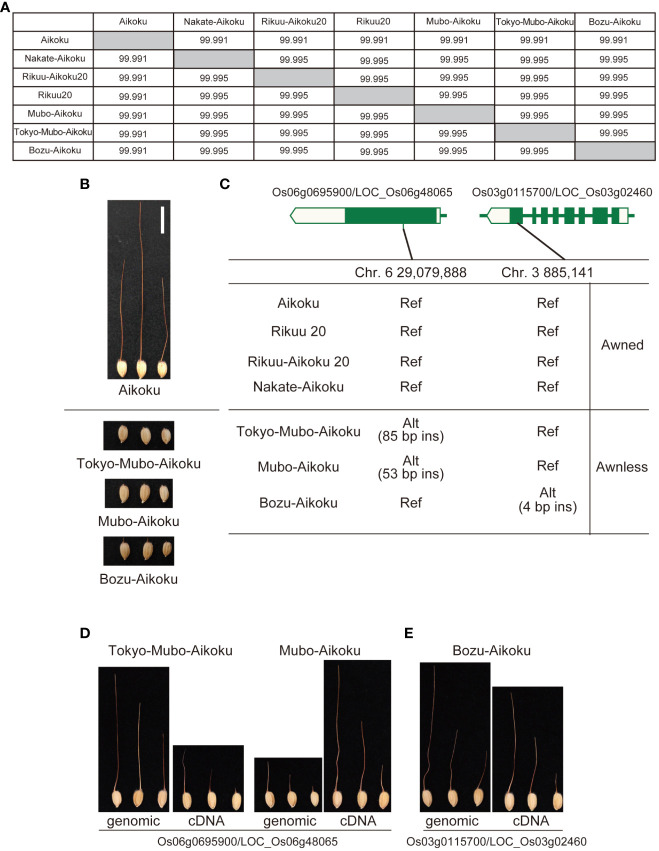
Identification of genes for awn formation from Aikoku varieties by comparison of their whole genomes. **(A)** Genomic similarity among four long-awned cultivars (Aikoku, Rikuu 20, Rikuu-Aikoku 20 and Nakate-Aikoku) and three awnless cultivars (Tokyo-Mubo-Aikoku, Mubo-Aikoku and Bozu-Aikoku). **(B)** Typical awned cultivar Aikoku and three awnless cultivars. **(C)** Exon-intron structure of Os06g0695900/LOC_Os06g48065 and Os03g0115700/LOC_Os03g02460 with DNA polymorphisms causing frameshift. **(D)** Tokyo-Mubo-Aikoku and Mubo-Aikoku with awns produced by transgenic complementation of whole genomic regions and cDNA of Os06g0695900/LOC_Os06g48065. **(E)** Bozu-Aikoku with awns produced by transgenic complementation of whole genomic regions and cDNA of Os03g0115700/LOC_Os03g02460. Alt, alternative allele; ins, insertion; Ref, reference allele.

Okada (1919) reported that mutation breeding had been carried out in Japan to remove the awn from Aikoku and its awnless mutants had been isolated. Based on this old document, we compared four long awned Aikoku with the three awnless varieties, which have almost identical genomes (**Figure 4**). There were no polymorphisms common to the three awnless varieties, but there are nine polymorphisms shared by the two mutants, Tokyo-Mubo-Aikoku and Mubo-Aikoku (**Supplementary Table 3**), while eight are specific to another mutant, Bozu-Aikoku (**Supplementary Table 4**), and we predicted that these are candidates for causative polymorphisms. Based on a comprehensive evaluation, DNA insertions expected to cause frameshift in Os06g0695900/LOC_Os06g48065 annotated as E3 ubiquitin ligase (53-bp insertion for Tokyo-Mubo-Aikoku and 85-bp insertion for Mubo-Aikoku) and a 4-bp DNA insertion expected to cause frameshift in Os03g0115700/LOC_Os03g02460, Short-chain dehydrogenase/reductase SDR family protein, for Bozu-Aikoku were selected (**Figure 4C**, **Supplementary Figure 3**). When the expression patterns of these genes were compared with known awn-forming genes (*An-1/RAE1*, Luo et al., 2013 and *An-2/LABA1*, Gu et al., 2015), they were found to share a common feature in that their expression is strongly repressed by ABA and jasmonic acid (**Supplementary Figures 4**, **5**). We also found a similarity in their expression is observed in the lemma/palea, pistil, and inflorescence (**Supplementary Figures 4**, **5**), supporting our findings.

To confirm that these genes are indeed involved in awn formation, we expressed the whole genomic regions of both genes or cDNAs under the control of the maize ubiquitin promoter in Tokyo-Mubo-Aikoku/Mubo-Aikoku or Bozu-Aikoku plants, respectively. As a result, all these transformants formed a long awn (**Figures 4D, E**). These results confirmed that both genes have functions essential for awn formation.

## 4 Discussion

In this study, we proposed an *in silico* approach that compares the whole genome sequence of a spontaneous mutant line with that of closely related lines and successfully identified four causative genes. These results indicate that this approach is effective in identifying genes that confer useful traits that can be used for breeding. The causative gene for dwarfism found in Ginbouzu-Miidashi and the gene for short grain found in Kamenoo-Daikoku were previously reported genes, respectively (Fujisawa et al., 1999; Itoh et al., 2004). On the other hand, of the two genes identified in this study, although Os06g0695900/LOC_Os06g48065 found in Tokyo-Mubo-Aikoku and Mubo-Aikoku is the same gene as *RAE3*, very recently identified in African rice (Bessho-Uehara et al., 2023), Os03g0115700/LOC_Os03g02460 found in Bozu-Aikoku is a novel gene involved in awn formation.

Previously, a method called “MutMap” was proposed as a way to map important agronomic traits using rice whole genome sequence information (Abe et al., 2012). This method can identify causative mutations by whole-genome sequencing of pooled DNA from segregants to reveal key genetic loci associated with important agronomic traits in rice, providing valuable insights for rice breeding and genetic improvement. In fact, the MutMap method has been successfully used to isolate many causative genes in several crop mutants. In addition, its improved methods have been proposed and their effectiveness has been reported in several cases (Fekih et al., 2013; Nordstrom et al., 2013; Sahu et al., 2020). However, these methods are essentially based on a genetic approach in which the mutant of interest is crossed with its parental line, the F2 generations are distinguished on the basis of phenotype, and the whole genome is compared between the two to find the mutation that causes the difference in trait. This requires at least two generations of cultivation and a reasonable number of individuals for the F2 segregation generation, which is time consuming and labor intensive. Such problems may not be a major obstacle in the case of model plants/crops such as Arabidopsis and rice, but they can be a major obstacle for plants that take a long time to update a generation, such as trees, perennial crops, some grasses that flower only once every few decades. In addition, many of these plants are often difficult to grow in large populations.

The method we propose in this study does not involve a genetic approach and therefore does not have any of the limitations of MutMap and its modified approaches. Instead, it may be difficult to apply this method widely and generally to many plants and crops. In fact, this method can only be applied to mutants produced by natural mutation. There are two sides to this feature - a good side and a bad side. On the positive side, as we have already mentioned, the natural mutation rate per generation is not high, and therefore it is possible to identify the gene responsible for the mutation by directly comparing the whole genome sequence without resorting to genetic methods, as shown in this paper. On the other hand, this fact clearly shows a disadvantage of this method: the spontaneous mutation rate is low and the resulting mutants are rare. However, in the case of rice, for example, this plant has been cultivated for a very long time as a very important crop, and during this time many mutants have accumulated due to natural mutation. For example, the mutant that produces Daikoku-type seeds was repeatedly introduced in various literature published in the 17th and 19th centuries (Sase, 1684, Iwasaki, 1828), and in fact many different mutant alleles have been found as Daikoku mutants (**Figure 3**, Fujisawa et al., 1999). In the case of plant species that have a long history of cultivation and many lines with abnormal phenotypes have been preserved (e.g. fruit trees and orchids), the approach proposed here can be expected to lead to the causal gene of the mutation with only a very low-cost and low-task whole genome analysis.

Theoretically, it is most likely to succeed by directly comparing the sequences between the plant with the mutant phenotype and its siblings without the mutant phenotype. However, in this study, since those siblings were not available, we used several varieties whose genomes were almost identical to the mutants. In this case, the number of mutations detected was higher than theoretical (**Figure 1**), but still the causal polymorphisms were successfully identified. Therefore, this method is applicable when genomically close varieties are available. It should be noted, however, that this approach will not always be successful, for example, when the mutation alters gene expression or has epigenetic effects. However, even in such cases, it is possible to predict candidate genes by searching for causative polymorphisms in the promoter regions and comparing their level and specificity of expression.

In terms of mutation rates, a large study of *de novo* mutations in Arabidopsis has recently been reported (Monroe et al., 2022). According to this study, contrary to the common theory (mutagenesis occurs randomly across the genome), genes under strong selective pressure have a low mutation frequency. If this is true for crops, the mutation rate could vary depending on the trait of interest. In any case, the mutation rate at the whole genome level in plants, especially in crops that are always under artificial control, needs to be analyzed in the future.

Although there are many lines that have been produced by spontaneous mutation in the past, only a few of them have been used in breeding programs, leaving a large amount of unutilized genetic resources. The *in silico* approach proposed in this study will enable the identification of useful genes that can be used for crop breeding with minimal effort required for NGS analysis. Effective use of this approach will accelerate the molecular breeding through such as the generation of molecular markers (Hasan et al., 2021) and the breeding of new varieties through pinpoint improvement using the latest genome editing technologies (Mishra et al., 2018).

## Data availability statement

The datasets presented in this study can be found in online repositories. The names of the repository/repositories and accession number(s) can be found in the article/**Supplementary Material**.

## Author contributions

MS: Data curation, Formal Analysis, Investigation, Methodology, Software, Validation, Visualization, Writing – original draft, Writing – review & editing. SK: Data curation, Formal Analysis, Investigation, Validation, Visualization, Writing – original draft, Writing – review & editing. HY: Data curation, Formal Analysis, Investigation, Methodology, Resources, Software, Validation, Visualization, Writing – original draft, Writing – review & editing. MMo: Data curation, Formal Analysis, Investigation, Resources, Writing – original draft. MK: Data curation, Formal Analysis, Investigation, Resources, Writing – original draft. EK: Data curation, Formal Analysis, Investigation, Resources, Writing – original draft. MMa: Conceptualization, Data curation, Formal Analysis, Funding acquisition, Investigation, Methodology, Project administration, Resources, Supervision, Validation, Visualization, Writing – original draft, Writing – review & editing.
