# Supplementary material for: Low mutation rate of spontaneous mutants enables detection of causative genes by comparing whole genome sequences
Source: Front Plant Sci. 2024 Apr 4;15:1366413. doi: 10.3389/fpls.2024.1366413 (PMC11024370; doi:10.3389/fpls.2024.1366413)
Supplement: Supplementary file 1 [file DataSheet_1.pdf]

```
Os06t0570100-01           --------------------------MEAFVPGGAGAAAAAVGGFVAAAALAERAGVIAP
AT5G25900.1               -----------------------MAFFSMISILLGFVISSFIFIFFFKKLLSFSRKNMSE
Glyma.13G371400.1.p       -----------------------------MHAMEFLTLSVTVAAAAFSILFFFLRHAGAG
Solyc04g083160.1.1        -------------------MDAILNLQGVPLGTALTVGGPVVALGGISFWLLKEYVNDQK
GRMZM2G059308_P01         -----------------------MESLVAALPAGGAAAAAAFGGLVAAAALAGKVGLVGS
Sobic.010G172700.1.p      -----------------------MESLVAALPAGGAAAAAAVGGLVAAAALAGKAGLVGP
Si006269m                 -------------------METMLASLVPASGTAAAAAAAAGGLVAAAALVAGGGRLVGQ
Pavir.J31452.1.p          -----------------------MESLVAALPTGSAAAAAAVGGLLTAAALAGRAGAKNN
74427                     ------------------------------------------------------------
Pp3c20_800V3.1.p          MAKHLATQLLQQWNEALKTMPPGFRTAGKILVWEELASNKVLITIALAWVLLFVARTCLR

Os06t0570100-01           RKRPNAPPAVPGLPIIGNLHQLKEKKPHQTFAKWAEIYGPIYTIRTGASSVVVLNSTEVA
AT5G25900.1               VSTLPSVPVVPGFPVIGNLLQLKEKKPHKTFTRWSEIYGPIYSIKMGSSSLIVLNSTETA
Glyma.13G371400.1.p       AGSLPPVPAVPGLPVIGNLLQLKEKKPYKTFTQMAHKHGPIYSIRTGASTLIVLNSPLLA
Solyc04g083160.1.1        RKSPNFLPPVPGLPVIGNLLQMTEKKPHKTFTNWAETYGPIYSIKTGANTIVVLSSSELA
GRMZM2G059308_P01         KKHLNAPPAVSGLPLIGNLHQLKEKKPHQTFTKWAEIYGPIYTIRTGSSTVVVLNSAQVA
Sobic.010G172700.1.p      KKHLNAPPAVPGLPMIGNLHQLKEKKPHQTFTKWAEIYGPLYTIRTGASSVVVLNSAQVA
Si006269m                 KDRLNAPPAVPGLPIIGNLHQLKERKPHQTFARWAQVYGPIYTIRTGASSVAVLNSTEVA
Pavir.J31452.1.p          R--STAPPAVPGLPIVGNLLQLKEKKPHQTFTKWAEIYGPIYTIKTGASSAVVLNSTEVA
74427                     ------VAEVPGLPFVGNLLQMTVERPHRKLTSWSNEYGPIYTIRTGQKSQVIVSSPELA
Pp3c20_800V3.1.p          NKKRLPPAIPGGLPVLGNLLQLTEKKPHRTFTAWSKEHGPIFTIKVGSVPQAVVNNSEIA
                                 .   *:*.:*** *:. .:*::.::  :. :**:::*: *  .  ::...  *

Os06t0570100-01           KEAMVAKFSSISTRKLSKALTVLTRDKSMVATSDYCDFHKMVKRYVMSSMLGTSAQKQFR
AT5G25900.1               KEAMVTRFSSISTRKLSNALTVLTCDKSMVATSDYDDFHKLVKRCLLNGLLGANAQKRKR
Glyma.13G371400.1.p       KEAMVTRFSSISTRKLSNALKILTSDKCMVATSDYNEFHKTVKRHILTNFLGANAQKRHH
Solyc04g083160.1.1        KEAMVTRYSSISTRKLTNALRILTCDKSIVAISDYDEFHKTAKRHILTSVLGPTAQKRFR
GRMZM2G059308_P01         KEAMIAKFSSISTRKLSKALSALTRDKTMVATSDYGDFHKMIKRYIMTFMLGTSGQKQFR
Sobic.010G172700.1.p      KEAMIAKFSSISTRKLSKALSVLTRDKTMVATSDYGDFHKMVKRYVMTSMLGTSAQKQFR
Si006269m                 KEAMVAKFSSISTRKLSNALSVLTCDKKIVATSDYGDFHKMVKRYVMVSMLGASSQKQFR
Pavir.J31452.1.p          KEAMIAKFSSISTRKLSKALSVLTHDKTMVATSDYGDFHKMVKRYIMTSMLGTSGQKKFR
74427                     REAVVAKYSSISNRDLGSNLTILTRNRKIVAMSDYGDRYRMLKRMVVNNLLGQTSQKALH
Pp3c20_800V3.1.p          KEVLVTKFASISKRQMPMALRVLTRDKTMVAMSDYGEEHRMLKKLVMTNLLGPTTQNKNR
                          :*.::::::***.*.:   *  ** :: :** *** : ::  *: ::  .** . *:  :

Os06t0570100-01           DIRDMMIHNMLSTFHKLVKDDPHAP--LIFRDVFKDELFRLS--MIQSLGEDVSSVYVDE
AT5G25900.1               HYRDALIENVSSKLHAHARDHPQEP--VNFRAIFEHELFGVA--LKQAFGKDVESIYVKE
Glyma.13G371400.1.p       IHREAMMENILSQFSEHVKTFSDLA--VNFRKIFVTQLFGLA--LKQALGSNVETIYVEE
Solyc04g083160.1.1        IHRDTLVENVSKQLHDLVRTDPNEA--VNLRKSFQSELFGLA--LKALG-KDIEAIYVEG
GRMZM2G059308_P01         DTRNMMVDNMLNTFHTLLMDDPNSP--LNFREVFKNELFRLS--LVQALGEDVSSIYVEE
Sobic.010G172700.1.p      DTRNMMVDNMLNTFHTLLTDDPNSP--LNFREVFKNELFRLS--LIQALGEDVSSVYVEE
Si006269m                 GIRDMVIDNMLSTFHTLVTDDPKAA--LNFREVFKNELFRLS--LIQALGEDMSSVYVEE
Pavir.J31452.1.p          DTRNMMVDNMLSTFHTLLNDDPNAP--LNFREVFTNELFRLS--LIQALGEDVSSVYVEE
74427                     VQRENYLGIALDGLFDELGRFPGSTGQVNARDCIANFLFRLGTHQASVFGRDIESVRVPE
Pp3c20_800V3.1.p          SLRDDALIGMIEGVLAELKASPTSPKVVNVRDYVQRSLFPFALQQV--FGYIPDQVEVLE
                            *:  :    . .       .  .  :  *  .   ** ..           . : *
                                                     S
Os06t0570100-01           FGRDISKEEIYNATVTDMMMCAIEVDWRDFFPYLSWVPNKSFETRVFTTETRRTAVMRAL
AT5G25900.1               LGVTLSKDEIFKVLVHDMMEGAIDVDWRDFFPYLKWIPNKSFEARIQQKHKRRLAVMNAL
Glyma.13G371400.1.p       LGSTLSKEDIYKILVVDIMEGAIEVDWRDFFPYLKWIPNRRLEMKIQNLYVRRKAVMKAL
Solyc04g083160.1.1        LDATLPREDLLNILVLDIMEGAIDVDWRDFFPYLKWVPNKSFEHRIQRKHLRREAVMKAL
GRMZM2G059308_P01         YGKVISKEEIYKATVVDMMMCAIEVDWRDFFPYLSWIPNRTFETRVLTTEARRTTVMQAL
Sobic.010G172700.1.p      YGKVISQEEIYQATVADMMMCAIEVDWRDFFPYLSWIPNRSFETRVLTTEARRTTVMQAL
Si006269m                 FGKSISKEEIYQTTVVDLMMCALAVDWRDFFPYLRWIPNRNFETKVSTTEARRTAVIRAL
Pavir.J31452.1.p          FGKDISKEEIYRATVVDMMMCAIEVDWRDFFPYLSWVPNRSFETRVLTTEGRRTAVMRAL
74427                     LGAEVTRWEIYRILVQDVMKAAVQIDWRDFFPTLKWIPNWKFEDGIYKVERKRSAVTKAL
Pp3c20_800V3.1.p          LGTCVSTWDMFDALVVAPLSAVINVDWRDFFPALRWIPNRSVEDLVRTVDFKRNSIMKAL
                           .  :.  ::    *   :  .: :******* * *:**  .*  :     :* :: .**

Os06t0570100-01           IKQQKERIVRGEAKT-CYLDFLLAEN-TLTDEQLMMLVWEALIEAADTTLVTTEWAMYEL
AT5G25900.1               IQDRLKQNGSESDDD-CYLNFLMSEAKTLTKEQIAILVWETIIETADTTLVTTEWAIYEL
Glyma.13G371400.1.p       MNEQKNRMASGKEVN-CYFDYLVSEAKELTEDQISMLIWETIIETSDTTLVTTEWAMYEL
Solyc04g083160.1.1        IADQRKRIISGEELN-SYIDYLLSEANTLTEKQILMLLWEAIIETSDTTLVSTEWAMYEL
GRMZM2G059308_P01         IKQQKERIARGETRI-SYLDFLLAEN-TLTDEQLLMLVWEAVIEAADTTLVTTEWAMYEI
Sobic.010G172700.1.p      INQQKIRIARGETRI-SYLDFLLAEN-TLTDEQLLMLVWEAVIEAADTTLVTTEWAMYEI
Si006269m                 INQQKKRIARGEARV-SYLDFLVAQN-TLTDEQVTTLVWEAVIEAADTTLVTTEWAMYEL
Pavir.J31452.1.p          INQQKKRIARGETRI-SHLDFLLAEN-TLNDEQLLMLVWEAVIEAADTTLVTTEWAMYEV
74427                     MEQHRQ-LSRSQQRDKCYCDVLLDNESHYSEDELLLAAWEPIIESSDTTLVTSEWALYEL
Pp3c20_800V3.1.p          IRAQRMRLANLKEPPRCYADIALTEATHLTEKQLEMSLWEPIIESADTTLVTSEWAMYEI
                          :  :       .    .: :  : :    ...::    **.:**::*****::***:**:
```

```
Os06t0570100-01          AKNPDKQERLYQEIREVCGDE-TVTEEHLPRLPYLNAVFHETLRRHSPVPLIPPRFVHED
AT5G25900.1              AKHPSVQDRLCKEIQNVCGGE-KFKEEQLSQVPYLNGVFHETLRKYSPAPLVPIRYAHED
Glyma.13G371400.1.p      AKDKTRQDRLYEELQYVCGHE-NVIEDQLSKLPYLGAVFHETLRKHSPAPIVPLRYAHED
Solyc04g083160.1.1       AKDSKRQEQLFLEIQNVCGSN-KITEEKLCQLPYLCAVFHETLRKYSPAPIVPLRYVHED
GRMZM2G059308_P01        AKHPEKQEYLYQEIQKVCGNK-TVTEDHLPELPYLNAVFHETMRRHSPVPLVPPRLVHEN
Sobic.010G172700.1.p     AKHPEKQEYLYQEIQKVCGNK-TVTEDHLPELPYLNAVFHETLRRHSPVPLVPPRFVHEN
Si006269m                CKNQEKQERLFEEIQEVCGDE-TVTEDDLPRLPYLNAVFHETLRRHPPVSLVPPRFVHEN
Pavir.J31452.1.p         AKHPEKQDRLYQEIQEVCGNE-MVTEDHLPQLPYLNAVFHETLRRHSPVPLVPPRYVHEN
74427                    ASAPKLQEKLYNEIKRVVGDERMVSEDDLPNLPFLNAVIKETLRKYSPVPILPPRYIHEQ
Pp3c20_800V3.1.p         AKNPDCQDRLYREIVSVAGTERMVTEDDLPNMPYLGAIIKETLRKYTPVPLIPSRFVEED
                         ..    *: *  *:  * * :  . *:.* .:*:* .:::**:*::.*..::* *  .*:

Os06t0570100-01          TKLAGYDVPAGTEMVINLYGCNMNRKEWESPEEWVPERFAG----GRLEVADMYKTMAFG
AT5G25900.1              TQIGGYHVPAGSEIAINIYGCNMDKKRWERPEDWWPERFLDD---GKYETSDLHKTMAFG
Glyma.13G371400.1.p      TKLGGYHIPAGSEIAINIYGCNMDNNLWENPNEWMPERFLD----EKYDHMDLYKTMAFG
Solyc04g083160.1.1       TQVGGYRIPKGTEIAINIYGCNRDKNVWESPEEWKPERFLN----GKYDPMELQKTMAFG
GRMZM2G059308_P01        TNLAGYEVPAGTEIIINLYGCNMNKNDWAEPEEWKPERFLD----GRFEAVDMHKTMAFG
Sobic.010G172700.1.p     TNLAGYEVPAGTEMIINLYGCNMNKSDWAEPEEWKPERFLD----GRFEAADMYKTMAFG
Si006269m                TTLAGYDIPAGTELIINLYGCNMNKNDWDEPEEWKPERFLD----GRFEQADMFKTMAFG
Pavir.J31452.1.p         TKLAGYVVPAGTEMVINLYGCNMNKSDWDEPEEWKPERFLD----GGFESADMYKTMAFG
74427                    VELGGYTIPAGYQLIVNIFGIHHDPKRWSNPETWDPSRFLGVEGGSFDMGLTDMRLMPFG
Pp3c20_800V3.1.p         ITLGGYDIPKGYQILVNLFAIANDPAVWSNPEKWDPERMLAN--KKVDMGFRDFSLMPFG
                           :.** :* * :: :*::.   :   *  *: * *.*:                 *.**

Os06t0570100-01          AGRRACAGSLQATHIACAAVARFVQEFGWRLREGDEEKVD-----TVQLTAYKLHPLHVH
AT5G25900.1              AGKRVCAGALQASLMAGIAIGRLVQEFEWKLRDGEEENVD-----TYGLTSQKLYPLMAI
Glyma.13G371400.1.p      AGKRVCAGSLQAMLIACTAIGRLVQQFEWELGQGEEENVD-----TMGLTTHRLHPLLVK
Solyc04g083160.1.1       AGKRVCAGAQQAMTISCTAIARLIQEFEWSLKEGEEKNVA-----TMGLTTHKLHPMLAH
GRMZM2G059308_P01        AGRRACAGSMQAMNISCTAIGRFVQEFAWRLEEGDEDKVD-----TIQLTTNRLYPLHVY
Sobic.010G172700.1.p     AGRRACAGSMQAMNISCTAIARFVQEFSWRLKEGDEDKAD-----TIQLTTNRLYPLHVY
Si006269m                AGRRACAGATQATNIACTAIARFVQDFAWRLKEGDEDKDD-----TIQLTTNRLYPLHVY
Pavir.J31452.1.p         AGRRACAGSLQALNISCTAIARFVQEFAWRLKEGDEDKAD-----TIHLTTNRLYPLYVY
74427                    GGKRICAGMAQVFYVVPMIIATLVQHFEWTLPQGDMDKRN-VVEDTVYLTTQKLEPLQAC
Pp3c20_800V3.1.p         AGKRMCAGITQAMFIIPMNVAALVQHCEWRLSPQEISNINNKIEDVVYLTTHKLSPLSCE
                         .*:* ***  *.  :    :. ::*.  * *   : .:       .  **: :* *:

Os06t0570100-01          LTRRGRM---------------------
AT5G25900.1              INPRRS----------------------
Glyma.13G371400.1.p      LKPRIK----------------------
Solyc04g083160.1.1       IKPRN-----------------------
GRMZM2G059308_P01        LAPRGRK---------------------
Sobic.010G172700.1.p     LTPRGRK---------------------
Si006269m                LTPRGRK---------------------
Pavir.J31452.1.p         LKPRGRK---------------------
74427                    AKPRVPRRLPSKTLNAVPSNNKVPKHKH
Pp3c20_800V3.1.p         ATPRISHRLP------------------
                            *
```

Supplemental Figure 1 Amino acid alignment of Os06g0570100

Mutations shown in yellow are thought to be the causative amino acid substitution for culm length (Itoh et al. 2004). Os06t0570100-01 (*Oryza sativa*), AT5G25900.1 (*Arabidopsis thaliana*), Glyma.13G371400.1.p (*Glycine max*), Solyc04g083160.1.1 (*Solanum lycopersicum* L.), GRMZM2G059308_P01 (*Zea mays*), Sobic.010G172700.1.p (*Sorghum bicolor*), Si006269m (*Setaria italica*), Pavir.J31452.1.p (*Paspalum virgatum*), 74427 (*Selaginella moellendorffii*), Pp3c20_800V3.1.p (*Physcomitrium patens*)
