# Supplementary material for: Low mutation rate of spontaneous mutants enables detection of causative genes by comparing whole genome sequences
Source: Front Plant Sci. 2024 Apr 4;15:1366413. doi: 10.3389/fpls.2024.1366413 (PMC11024370; doi:10.3389/fpls.2024.1366413)
Supplement: Supplementary file 2 [file DataSheet_2.pdf]

```
Os05t0333200-01          ------------------------------------------------------------
AT2G26300.1              ------------------------------------------------------------
Glyma.17G226700.1.p      ------------------------------------------------------------
Solyc08g061220.2.1       ------------------------------------------------------------
GRMZM2G064732_P02        ------------------------------------------------------------
Sobic.001G484200.1.p     ------------------------------------------------------------
Si022288m                ------------------------------------------------------------
Pavir.Ib00484.1.p        ------------------------------------------------------------
75723                    ------------------------------------------------------------
Pp3c1_38970V3.1.p        MAFPRNPQYSPTGEMKYRRRNEAGELDGSRAQPTTILGGQFGASTGGQASNGDDSMESSL

Os05t0333200-01          ------------------------------------------------------------
AT2G26300.1              ------------------------------------------------------------
Glyma.17G226700.1.p      ------------------------------------------------------------
Solyc08g061220.2.1       ------------------------------------------------------------
GRMZM2G064732_P02        ------------------------------------------------------------
Sobic.001G484200.1.p     ------------------------------------------------------------
Si022288m                ------------------------------------------------------------
Pavir.Ib00484.1.p        ------------------------------------------------------------
75723                    ------------------------------------------------------------
Pp3c1_38970V3.1.p        EGTSALTTREHLNGGGSSRIYSQFHNSNAEPEPSSVAARLKQQALDNPQQEQSVDDLLNS

Os05t0333200-01          ------------------------------------------------------------
AT2G26300.1              ------------------------------------------------------------
Glyma.17G226700.1.p      ------------------------------------------------------------
Solyc08g061220.2.1       ------------------------------------------------------------
GRMZM2G064732_P02        ------------------------------------------------------------
Sobic.001G484200.1.p     ------------------------------------------------------------
Si022288m                ------------------------------------------------------------
Pavir.Ib00484.1.p        ------------------------------------------------------------
75723                    ------------------------------------------------------------
Pp3c1_38970V3.1.p        IGVSLHANQKELDYSMAVDYGGPDKKDGFSKYKLSNSSHSGIKRCQTSDSIPVAVALSRI

Os05t0333200-01          ------------------------------------------------------------
AT2G26300.1              ------------------------------------------------------------
Glyma.17G226700.1.p      ------------------------------------------------------------
Solyc08g061220.2.1       ------------------------------------------------------------
GRMZM2G064732_P02        ------------------------------------------------------------
Sobic.001G484200.1.p     ------------------------------------------------------------
Si022288m                ------------------------------------------------------------
Pavir.Ib00484.1.p        ------------------------------------------------------------
75723                    ------------------------------------------------------------
Pp3c1_38970V3.1.p        GNKHYMSNKSRSIMQPSTQPQTNLSPPTVAPHASREGFVFPTLDKEVDTSIDLGSKSLEP

Os05t0333200-01          ------------------------------------------------------------
AT2G26300.1              ------------------------------------------------------------
Glyma.17G226700.1.p      ------------------------------------------------------------
Solyc08g061220.2.1       ------------------------------------------------------------
GRMZM2G064732_P02        ------------------------------------------------------------
Sobic.001G484200.1.p     ------------------------------------------------------------
Si022288m                ------------------------------------------------------------
Pavir.Ib00484.1.p        ------------------------------------------------------------
75723                    ------------------------------------------------------------
Pp3c1_38970V3.1.p        QEPSGNVSEVLDASIGDRSEEMAEPSHRMKLSSLESSQDISNGPFGVPSFSSAASTPVEM

Os05t0333200-01          ------------------------------------------------------------
AT2G26300.1              ------------------------------------------------------------
Glyma.17G226700.1.p      ------------------------------------------------------------
Solyc08g061220.2.1       ------------------------------------------------------------
GRMZM2G064732_P02        ------------------------------------------------------------
Sobic.001G484200.1.p     ------------------------------------------------------------
Si022288m                ------------------------------------------------------------
Pavir.Ib00484.1.p        ------------------------------------------------------------
75723                    ------------------------------------------------------------
Pp3c1_38970V3.1.p        SVAKGESVFDTPQQIPGEKIPKELVQGLQTDYPGDDEIESRTIDVPSFSVTTVDATIPSQ

Os05t0333200-01          ------------------------------------------------------------
AT2G26300.1              ------------------------------------------------------------
Glyma.17G226700.1.p      ------------------------------------------------------------
Solyc08g061220.2.1       ------------------------------------------------------------
GRMZM2G064732_P02        ------------------------------------------------------------
Sobic.001G484200.1.p     ------------------------------------------------------------
Si022288m                ------------------------------------------------------------
Pavir.Ib00484.1.p        ------------------------------------------------------------
75723                    ------------------------------------------------------------
Pp3c1_38970V3.1.p        VILQKSSSLHSGESSASVSPSLSVVLATTGLVANAPYTDESFNGEFNRSGADESSVMYSP

Os05t0333200-01          ------------------------------------------------------------
AT2G26300.1              ------------------------------------------------------------
Glyma.17G226700.1.p      ------------------------------------------------------------
Solyc08g061220.2.1       ------------------------------------------------------------
GRMZM2G064732_P02        ------------------------------------------------------------
Sobic.001G484200.1.p     ------------------------------------------------------------
Si022288m                ------------------------------------------------------------
Pavir.Ib00484.1.p        ------------------------------------------------------------
75723                    ------------------------------------------------------------
Pp3c1_38970V3.1.p        ESIQINSGFYTPKSYDSVEERSELVNIANHGGDRPAIPPVLPWKKGGCAGCGKGNLLLEK
```

```
Os05t0333200-01           ------------------------------------------------------------
AT2G26300.1               ------------------------------------------------------------
Glyma.17G226700.1.p       ------------------------------------------------------------
Solyc08g061220.2.1        ------------------------------------------------------------
GRMZM2G064732_P02         ------------------------------------------------------------
Sobic.001G484200.1.p      ------------------------------------------------------------
Si022288m                 ------------------------------------------------------------
Pavir.Ib00484.1.p         ------------------------------------------------------------
75723                     ------------------------------------------------------------
Pp3c1_38970V3.1.p         EYCMACGAKYCGSCVLQAMGSMPEGRKCISCLGQPIAGPRRPYIGKPSRLLRHLLSPLEV

Os05t0333200-01           ------------------------------------------------------------
AT2G26300.1               ------------------------------------------------------------
Glyma.17G226700.1.p       ------------------------------------------------------------
Solyc08g061220.2.1        ------------------------------------------------------------
GRMZM2G064732_P02         ------------------------------------------------------------
Sobic.001G484200.1.p      ------------------------------------------------------------
Si022288m                 ------------------------------------------------------------
Pavir.Ib00484.1.p         ------------------------------------------------------------
75723                     ------------------------------------------------------------
Pp3c1_38970V3.1.p         QQIMKAEKECPANQLRPEQVVVNNRPLSHEELAVLLGCQLPPGRLKPGKYWYDGQTGLWG

Os05t0333200-01           ------------------------------------------------------------
AT2G26300.1               ------------------------------------------------------------
Glyma.17G226700.1.p       ------------------------------------------------------------
Solyc08g061220.2.1        ------------------------------------------------------------
GRMZM2G064732_P02         ------------------------------------------------------------
Sobic.001G484200.1.p      ------------------------------------------------------------
Si022288m                 ------------------------------------------------------------
Pavir.Ib00484.1.p         ------------------------------------------------------------
75723                     ------------------------------------------------------------
Pp3c1_38970V3.1.p         KEGHRPDRIVTPSLKVGGNLQPNASNGKTQVFMNGRELGKLELKMLKFAGVNCVPNTLLW

Os05t0333200-01           -----------------------------MGSSCSRSHSLSEAETTKNAKSADIDRRILQ
AT2G26300.1               -----------------------------MGLLCSRSR-HHTEDTDENTQAAEIERRIEQ
Glyma.17G226700.1.p       ---------------------MLSFVTENMGLLCSRNRRYNDADAEENAQTAEIERRIEL
Solyc08g061220.2.1        -----------------------------MGSLCSRNKHYSQADDEENTQTAEIERRIEQ
GRMZM2G064732_P02         -------------------MSVLTCVIESMGSSCSRSHSFDEAEAAENAKSADIDRRILQ
Sobic.001G484200.1.p      -------------------MSVLTCVIESMGSSCSRSHSLDETEAAENAKSADIDRRILQ
Si022288m                 -------------------MSVLTCVIESMGSSCSRHHSLNEAEAAENAKSADIDRRILQ
Pavir.Ib00484.1.p         -------------------MSVLTCVIESMGSSCSRHHSLNEAEATENAKSADIDRRILH
75723                     ----------------------------------------------------ELTRQLKQ
Pp3c1_38970V3.1.p         VEHDGSYSEEGFKNVKGNIWAKEGNLWEKASIKLLYPFFSLPTPRSTNVNDCERHSNRFL
                                                                              :   .
                                                    L
Os05t0333200-01           ETKAEQHIHKLLLLGAGESGKSTIFKQIKLLFQTGFDEAELRSYTSVIHANVYQTIKILY
AT2G26300.1               EAKAEKHIRKLLLLGAGESGKSTIFKQIKLLFQTGFDEGELKSYVPVIHANVYQTIKLLH
Glyma.17G226700.1.p       ETKAEKHIQKLLLLGAGESGKSTIFKQIKLLFQTGFDEAELKSYLPVIHANVYQTIKLLH
Solyc08g061220.2.1        ETKAEKHIQKLLLLGAGDSGKSTIFKQIKLLFQTGFDEEELKNYIPVIHANVYQTIKILH
GRMZM2G064732_P02         ETKAEQHIHKLLLLGAGESGKSTIFKQIKLLFQTGFDEAELRSYTSVIHANVYQTIKILY
Sobic.001G484200.1.p      ETKAEQHIHKLLLLGAGESGKSTIFKQIKLLFQTGFDEAELKSYTSVIHANVYQTIKILY
Si022288m                 ETKAEQHIHKLLLLGAGESGKSTIFKQIKLLFQTGFDEAELRSYTSVIHANVYQTIKILY
Pavir.Ib00484.1.p         ETKAEQHIHKLLLLGAGESGKSTIFKQIKLLFQTGFDETELRSYTSVIHANVYQTIKILY
75723                     EDRIEKNVYKLLLLGSGESGKSTIFKQIKLLYNTGFGVEELKNYTPVIHANVYQAIKILY
Pp3c1_38970V3.1.p         GLADPLKVHKLLLLGHEGSGRSTIFKQAKILYNDGFTPEEYASFKTLIQANIYKMAILL
                                ::  ******   **:****** *:*:: **   *  .: .:*:**:*: : :*

Os05t0333200-01           EGAKELSQVE-----------------------------SDSSKYVISPDNQEIGEKLSD
AT2G26300.1               DGTKEFAQNE-----------------------------TDSAKYMLSSESIAIGEKLSE
Glyma.17G226700.1.p       DGSKEFAQND-----------------------------VDSSKYVISNENKEIGEKLLE
Solyc08g061220.2.1        DGSKELAQNE-----------------------------LEASKYLLSAENKEIGEKLSE
GRMZM2G064732_P02         EGAKELAQVE-----------------------------PDSSKYVLSPDNQEIGEKLSE
Sobic.001G484200.1.p      EGAKELAQVE-----------------------------PDSSKYVLSPDSQEIGEKLSE
Si022288m                 DGAKELAQVE-----------------------------PDSSKYVLSPDNQEIGEKLSE
Pavir.Ib00484.1.p         DGAKELAQVE-----------------------------PDSSKYVLSPDNQEIGEKLSE
75723                     EGCLDLQKKD-----------------------------VSG-EYTMRRENMEHGKRIAE
Pp3c1_38970V3.1.p         EGREHFEEEDDELANVSAKNHLDDTSVVSKENGDPSSSQPDAARFSGRNDENQKPKRSST
                          :*  .: : :                              .. .:    :.    ::

Os05t0333200-01           IDGRLDYPLLNKEL---------------------------VLDVKRLWQDPAIQETYLR
AT2G26300.1               IGGRLDYPRLTKDI---------------------------AEGIETLWKDPAIQETCAR
Glyma.17G226700.1.p       IGGRLDYPYLSKEL---------------------------AQEIENLWKDPAIQETYAR
Solyc08g061220.2.1        IGGRLDYPHLTKDL---------------------------VQDIEALWKDPAIQETLLR
GRMZM2G064732_P02         IGARLEYPSLNKER---------------------------VQDVRKLWQDPAIQETYSR
Sobic.001G484200.1.p      IGVRLDYPSLNKEC---------------------------VQDVRKLWQDPAIQETYSR
Si022288m                 IGAKLDYPLLNKEL---------------------------VQDVRKLWQDPAIQETYSR
Pavir.Ib00484.1.p         IGARLDYPLLNKEL---------------------------VQDVRKLWQDPAIQETYSR
75723                     IGDGVDYHPIGLLE---------------------------SDLIAQIWSDPAIQATYRK
Pp3c1_38970V3.1.p         GDNIYRLEPNLKELSDWFMDNMVCGNLESMSTSTSAHLQDHASKIEQLWLSSAVQATFQR
                           .                                          :  :* ..*:* *  :
```

```
Os05t0333200-01          GS-ILQLPDCAQYFMENLDRLAEAGYVP--TKEDVLYARVRTNGVVQIQFSPVGE-----
AT2G26300.1              GN-ELQVPDCTKYLMENLKRLSDINYIP--TKEDVLYARVRTTGVVEIQFSPVGE-----
Glyma.17G226700.1.p      GS-ELQIPDCTDYFMENLQRLSDANYVP--TKEDVLYARVRTTGVVEIQFSPVGE-----
Solyc08g061220.2.1       GN-ELQVPDCAHYFMENLERFSDVHYIP--TKEDVLFARIRTTGVVEIQFSPVGE-----
GRMZM2G064732_P02        GS-ILQVPDCAQYFMENLDKLSEEDYVP--TKEDVLHARVRTNGVVETQFSPLGE-----
Sobic.001G484200.1.p     GS-ILQVPDCAQYFMENLDRLSEVDYVP--TKEDVLHARVRTNGVVETQFSPLGE-----
Si022288m                GS-ILQVPDCAQYFMSNLDRLAEVDYVP--TKEDVLHARVRTNGVVETQFSPLGE-----
Pavir.Ib00484.1.p        GS-ILQVPDCAQYFMNNLDRLAEVNYLP--TKEDVLHARVRTNGVVEIQFSPLGE-----
75723                    AN-ELQLPDCTEYFLSGVDRLAKPDYIPTEACEDILHARVRTTGIADVVFKHDG------
Pp3c1_38970V3.1.p        RSELPFLPDLASFFLNRVVDVCRSDYEPAEIDILRTEGLSQGSGLVQIEITLDDNPGSWQ
                          .    :** : :::. :  ..   * *         .  : .*:.:  :.  .

Os05t0333200-01          ---NKRGGEVYRLYDVGGQ-RNERRKWIHLFEGVNAVIFCAAISEYDQMLFEDETK--NR
AT2G26300.1              ---NKKSGEVYRLFDVGGQ-RNERRKWIHLFEGVTAVIFCAAISEYDQTLFEDEQK--NR
Glyma.17G226700.1.p      ---NKKSGEVYRLFDVGGQ-RNERRKWIHLFEGVSAVIFCAAISEYDQTLFEDENR--NR
Solyc08g061220.2.1       ---NKKSGEVYRLFDVGGQ-RNERRKWIHLFEGVTAVIFCAAISEYDQTLFEDERK--NR
GRMZM2G064732_P02        ---SKRGGEVYRLYDVGGQ-RNERRKWIHLFEGVNAVIFCAAISEYDQMLFEDETK--NR
Sobic.001G484200.1.p     ---SKRGGEVYRLYDVGGQ-RNERRKWIHLFEGVNAVIFCAAISEYDQMLCEDETK--NR
Si022288m                ---SKRGGEVYRLYDVGGQ-RNERRKWIHLFEGVNAVIFCAAVSEYDQMLFEDETK--NR
Pavir.Ib00484.1.p        ---SKRGGEVYRLYDVGGQ-RNERRKWIHLFEGVNAVIFCVAISEYDQVLFEDETK--NR
75723                    --------HTYRVFDVGGQ-RNERRKWLHLFDGVKAVIFCAALSEYDQNLFEDEGK--NR
Pp3c1_38970V3.1.p        DVDTTTGLDRYQVIRVGGKGMSDRHKWLDMFEDVRAVVFCVALSDYNSLWNDRSGNPSNK
                                 . *::  ***:  .:*:**:.:*:.* **:**.*:*:*:.   : . .  *:

Os05t0333200-01          MMETKELFDWVLKQRCFEKTSFILFLNKFDIFEKKIQK-VPLSVCEWFKDYQPIAPGKQ-
AT2G26300.1              MMETKELFDWVLKQPCFEKTSFMLFLNKFDIFEKKVLD-VPLNVCEWFRDYQPVSSGKQ-
Glyma.17G226700.1.p      MMETKELFEWILKQPCFEKTSFMLFLNKFDIFEKKILK-VPLNVCEWFKDYQPVSTGKQ-
Solyc08g061220.2.1       MMETKELFEWVLKQPCFEKTSFMLFLNKFDIFEQKVPK-VPLNACEWFKDYQSVSTGKQ-
GRMZM2G064732_P02        MMETKELFDWVLKQRCFEKTSFMLFLNKFDIFERKIQK-VPLSVCEWFKDYQPTAPGKQ-
Sobic.001G484200.1.p     MMETKELFDWVLKQRCFEKTSFMLFLNKFDIFERKIQK-VPLSACEWFKDYQPIAPGKQ-
Si022288m                MMETKELFDWVLKQRCFEKTSFMLFLNKFDIFERKIQK-VPLSVCEWFKDYQPTAPGKQ-
Pavir.Ib00484.1.p        MMETKELFDWVLKQRCFEKTSFMLFLNKFDIFERKIQK-VPLSVCEWFKDYQPTAPGKQ-
75723                    MVETMELFESVLRHPSFEKTSFLVFLNKYDIFRKKVLS-VPLNVCEVFRDYNEVQGDQER
Pp3c1_38970V3.1.p        MIQTRDLFESILRHPCFQDTPFVLLLNKYDVFEDKIEQGVPLTTCTWFSDFRPVGTSHYT
                         *::* :**: :*:: .*:.*.*:::***:*:*. *: . ***..*  * *:.    .:

Os05t0333200-01          EVEHAYEFVKKKFEELYFQSSKPDRVDRVFKIYRTTALDQKLVKKTFKLIDESMRRSREG
AT2G26300.1              EIEHAYEFVKKKFEELYYQNTAPDRVDRVFKIYRTTALDQKLVKKTFKLVDETLRRRNLL
Glyma.17G226700.1.p      EIEHAYEFVKKKFEESYFQSTAPDRVDRVFKIYRTTALDQKVVKKTFKLVDETLRRRNLL
Solyc08g061220.2.1       EIEHAYEFVKKKFEESYFQCTAPDRVDRVFKIYRTTALDQKLVKKTFKLVDETLRRRNLF
GRMZM2G064732_P02        EVEHAYEFVKKKFEELYFQSSKPDRVDRVFKIYRTTALDQKLVKKTFKLIDESMRRSREG
Sobic.001G484200.1.p     EVEHAYEFVKKKFEELYFQSSKPDRVDRVFKIYRTTALDQKLVKKTFKLIDESMRRSREG
Si022288m                EVEHAYEFVKKKFEELYFQSSKPDRVDRVFKIYRTTALDQKLVKKTFKLIDESMRRSREG
Pavir.Ib00484.1.p        EVEHAYEFVKKKFEELYFQSSKPDRVDRVFKIYRTTALDQKLVKKTFKLIDESMRRSREG
75723                    KISHALQYIKNKFDEIYKRNTPGLGTQRLCWLFETTALDPRIMKYTFELVDKNLVVSSIS
Pp3c1_38970V3.1.p        AQNQAQQAYQYIAHKYKELFNSVDCTGRKLFTFQLNALDKTTVSGAFNYVKQILKWDEQK
                           .:* :  :   .:     .    . *    :. .***   :. :*: :.: :

Os05t0333200-01          T-----------------------------------------
AT2G26300.1              EAGLL-------------------------------------
Glyma.17G226700.1.p      EAGLL-------------------------------------
Solyc08g061220.2.1       EAGLL-------------------------------------
GRMZM2G064732_P02        T-----------------------------------------
Sobic.001G484200.1.p     T-----------------------------------------
Si022288m                T-----------------------------------------
Pavir.Ib00484.1.p        T-----------------------------------------
75723                    LL----------------------------------------
Pp3c1_38970V3.1.p        AAGWGIIPDEMSSYSTDISSFSRHSSHSMTRQPDLFRRQHHY
```

Supplemental Figure 2 Amino acid alignment of Os05g0333200

Mutations shown in yellow are thought to be the causative amino acid substitution for short grain in this study. Previous work indicated that this is the causative gene for short grain with different mutation sites (Fujisawa et al. 1999). Os05t0333200-01 (*Oryza sativa*), AT2G26300.1 (*Arabidopsis thaliana*), Glyma.17G226700.1.p (*Glycine max*), Solyc08g061220.2.1 (*Solanum lycopersicum* L.), GRMZM2G064732_P02 (*Zea mays*), Sobic.001G484200.1.p (*Sorghum bicolor*), Si022288m (*Setaria italica*), Pavir.Ib00484.1.p (*Paspalum virgatum*), 75723 (*Selaginella moellendorffii*), Pp3cl_38907V3.1.p (*Physcomitrium patens*)
