# Supplementary material for: Low mutation rate of spontaneous mutants enables detection of causative genes by comparing whole genome sequences
Source: Front Plant Sci. 2024 Apr 4;15:1366413. doi: 10.3389/fpls.2024.1366413 (PMC11024370; doi:10.3389/fpls.2024.1366413)
Supplement: Supplementary file 3 [file DataSheet_3.pdf]

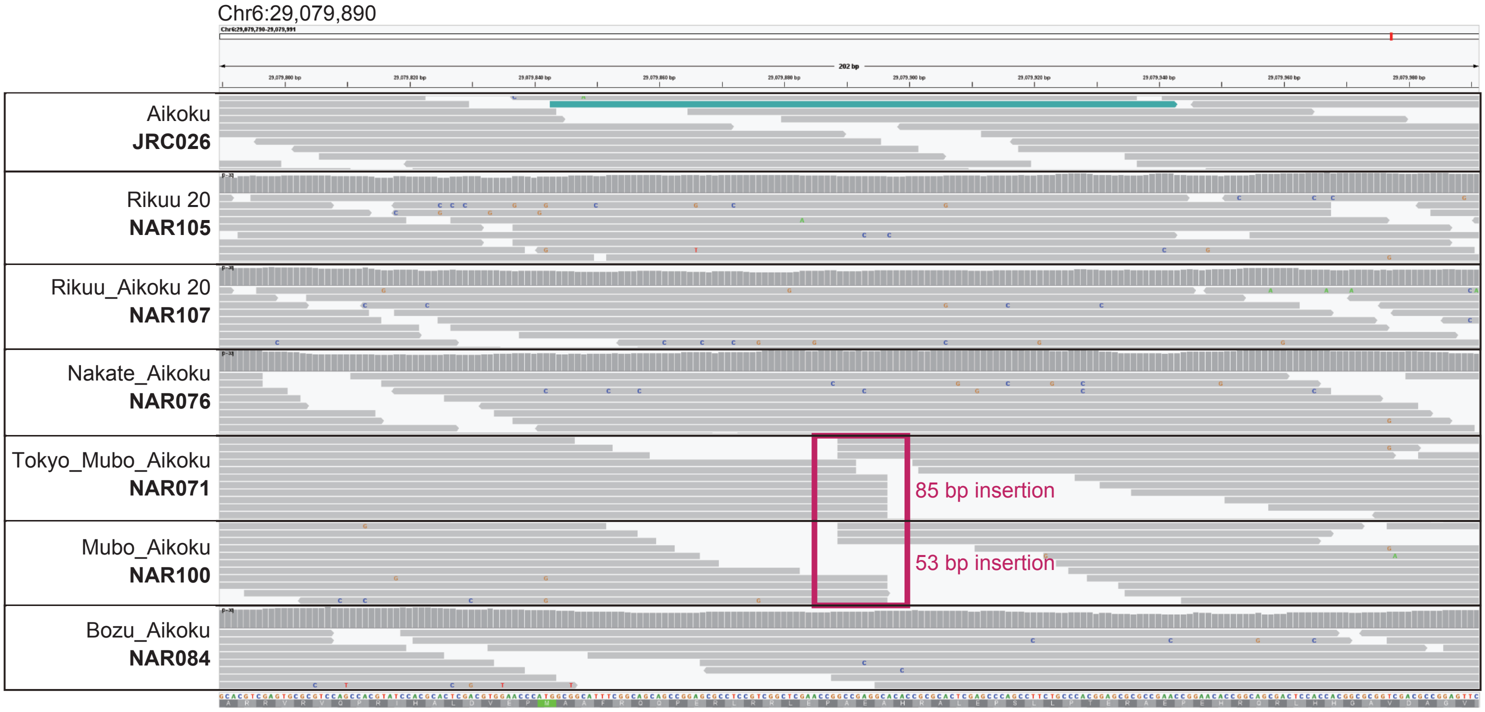
Chr6:29,079,890
Aikoku
JRC026
Rikuu 20
NAR105
Rikuu_Aikoku 20
NAR107
Nakate_Aikoku
NAR076
Tokyo_Mubo_Aikoku
NAR071
85 bp insertion
Mubo_Aikoku
NAR100
53 bp insertion
Bozu_Aikoku
NAR084

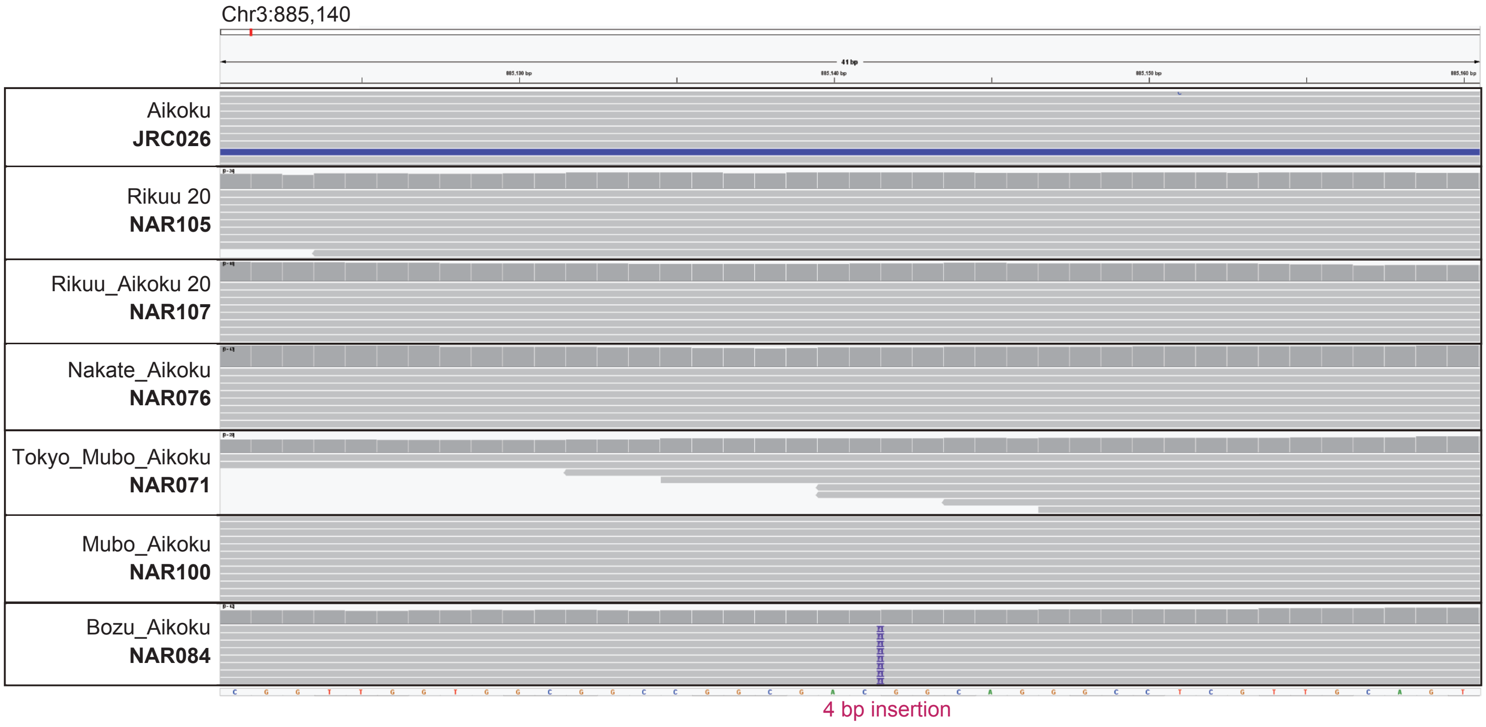
Chr3:885,140
Aikoku
JRC026
Rikuu 20
NAR105
Rikuu_Aikoku 20
NAR107
Nakate_Aikoku
NAR076
Tokyo_Mubo_Aikoku
NAR071
Mubo_Aikoku
NAR100
Bozu_Aikoku
NAR084
4 bp insertion

Supplemental Figure 3 Integrative Genomics Viewer (IGV) image of Aikoku and its derivatives

(A) The insertion mutations were found in the genomic region of Os06g0695900/LOC_Os06g48065. (Tokyo-Mubo-Aikoku has 85 bp insertion mutation, while Mubo-Aikoku has 53 bp insertion mutation. (B) The 4 bp insertion mutation found in the genomic region of Os03g0115700/LOC_Os03g02460 in Bozu-Aikoku.
