# Supplementary material for: Low mutation rate of spontaneous mutants enables detection of causative genes by comparing whole genome sequences
Source: Front Plant Sci. 2024 Apr 4;15:1366413. doi: 10.3389/fpls.2024.1366413 (PMC11024370; doi:10.3389/fpls.2024.1366413)
Supplement: Supplementary file 4 [file DataSheet_4.pdf]

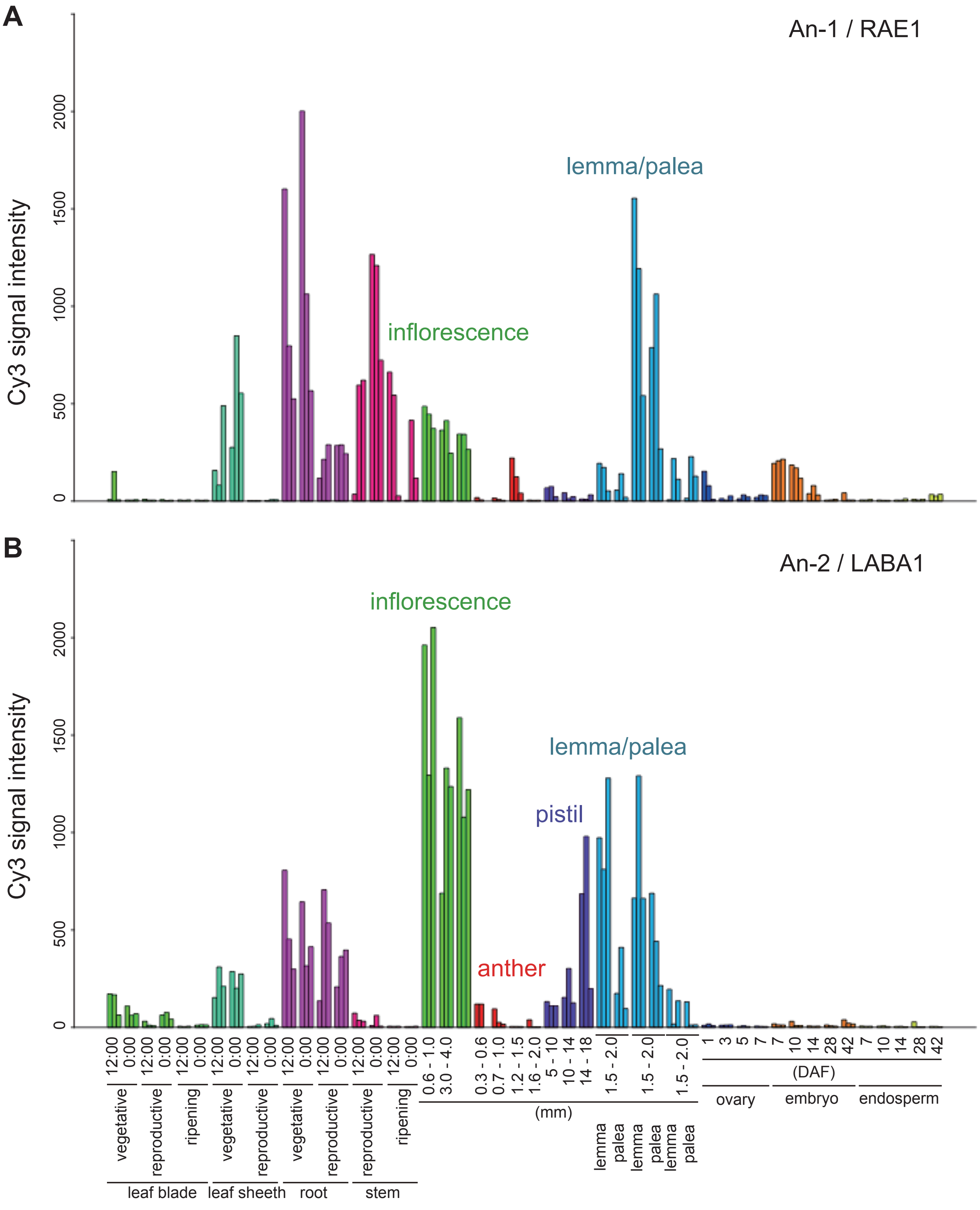
A
An-1 / RAE1
Cy3 signal intensity
0
500
1000
1500
2000
inflorescence
lemma/palea
B
An-2 / LABA1
Cy3 signal intensity
0
500
1000
1500
2000
inflorescence
lemma/palea
pistil
anther
12:00
0:00
vegetative
reproductive
ripening
leaf blade
leaf sheeth
root
stem
0.6 - 1.0
3.0 - 4.0
0.3 - 0.6
0.7 - 1.0
1.2 - 1.5
1.6 - 2.0
5 - 10
10 - 14
14 - 18
1.5 - 2.0
(mm)
lemma
palea
1
3
5
7
10
14
28
42
(DAF)
ovary
embryo
endosperm

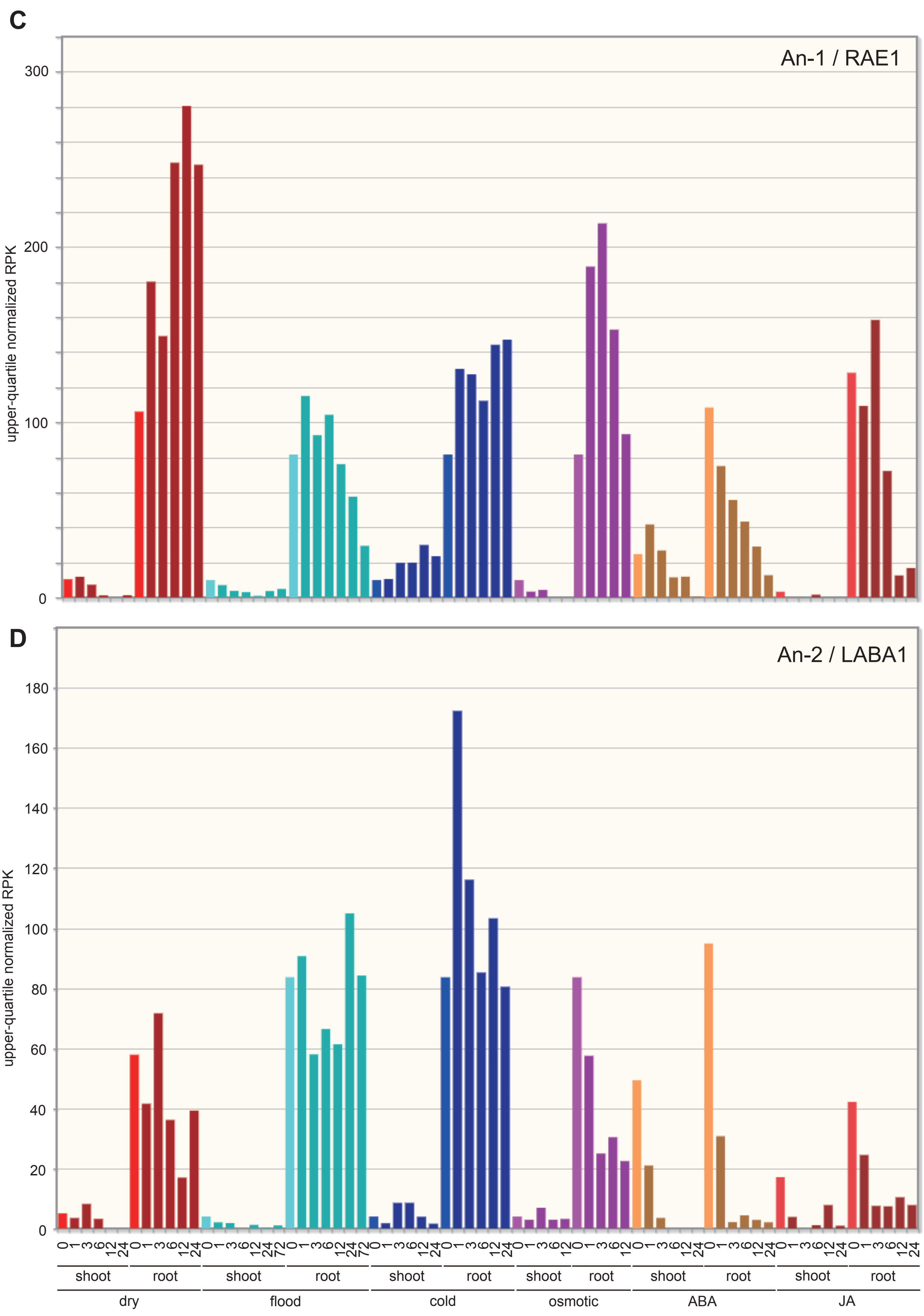
C
An-1 / RAE1
upper-quartile normalized RPK
0
100
200
300
D
An-2 / LABA1
upper-quartile normalized RPK
0
20
40
60
80
100
120
140
160
180
0 1 3 6 12 24 0 1 3 6 12 24
0 1 3 6 12 24 72 0 1 3 6 12 24 72
0 1 3 6 12 24 0 1 3 6 12 24
0 1 3 6 12 0 1 3 6 12
0 1 3 6 12 24 0 1 3 6 12 24
0 1 3 6 12 24 0 1 3 6 12 24
shoot
root
dry
flood
cold
osmotic
ABA
JA

**Supplemental Figure 4 The expression of known awn genes An-1/RAE1 and An-2/LABA1**

The expression of An-1/RAE1 (A and C) and An-2/LABA1 (B and D). Temporal and spatial expression was investigated in Rice X pro, while the expression under abiotic stress condition was searched in TENOR (Transcriptome ENcyclopedia Of Rice) database. DAF, days after flowering; ABA, abscisic acid; JA, jasmonic acid.
