# Supplementary material for: Low mutation rate of spontaneous mutants enables detection of causative genes by comparing whole genome sequences
Source: Front Plant Sci. 2024 Apr 4;15:1366413. doi: 10.3389/fpls.2024.1366413 (PMC11024370; doi:10.3389/fpls.2024.1366413)
Supplement: Supplementary file 5 [file DataSheet_5.pdf]

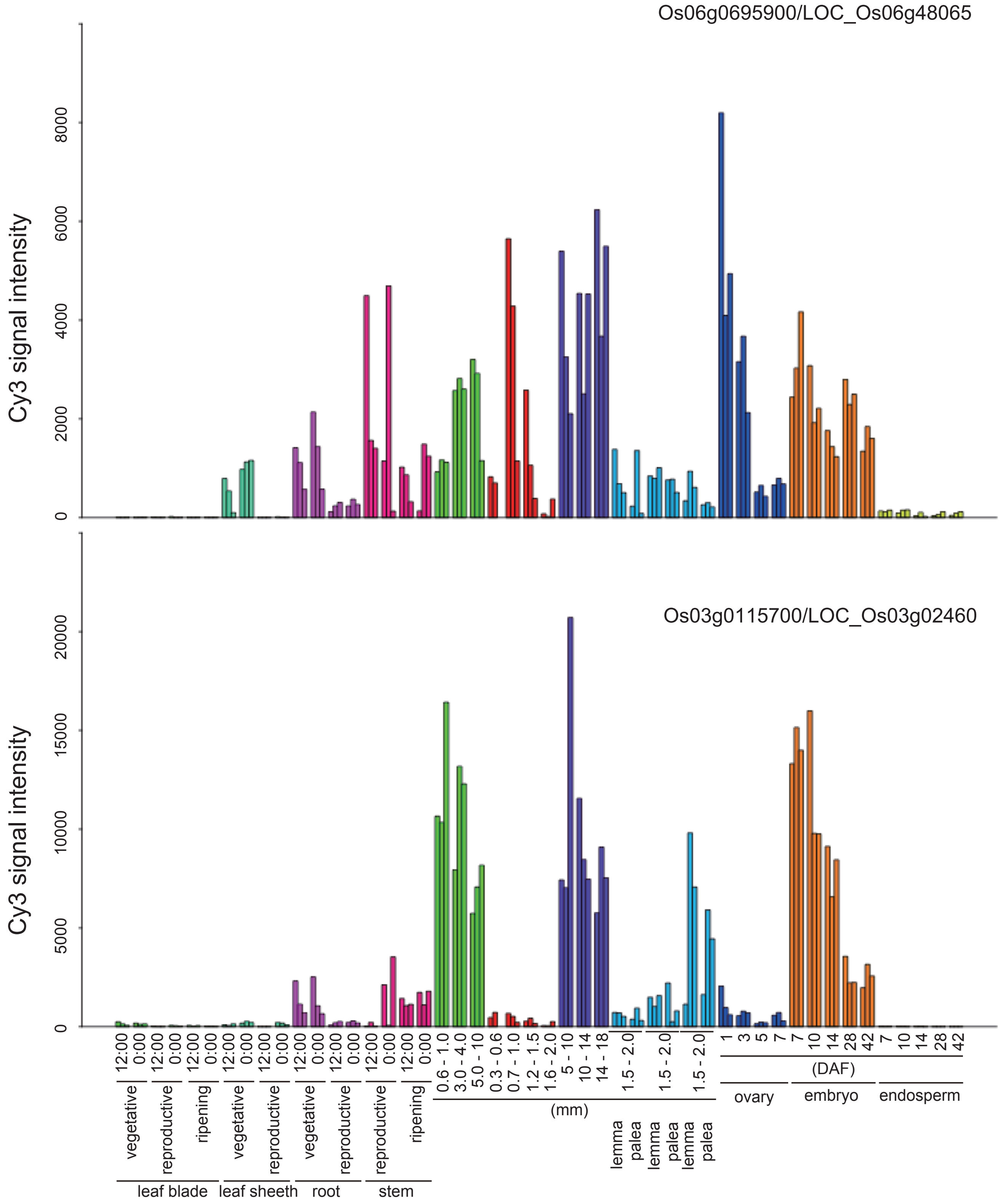

Os06g0695900/LOC_Os06g48065
Cy3 signal intensity
0
2000
4000
6000
8000
Os03g0115700/LOC_Os03g02460
Cy3 signal intensity
0
5000
10000
15000
20000
12:00
0:00
vegetative
reproductive
ripening
leaf blade
leaf sheeth
root
stem
0.6 - 1.0
3.0 - 4.0
5.0 - 10
0.3 - 0.6
0.7 - 1.0
1.2 - 1.5
1.6 - 2.0
5 - 10
10 - 14
14 - 18
(mm)
1.5 - 2.0
lemma
palea
1
3
5
7
10
14
28
42
(DAF)
ovary
embryo
endosperm

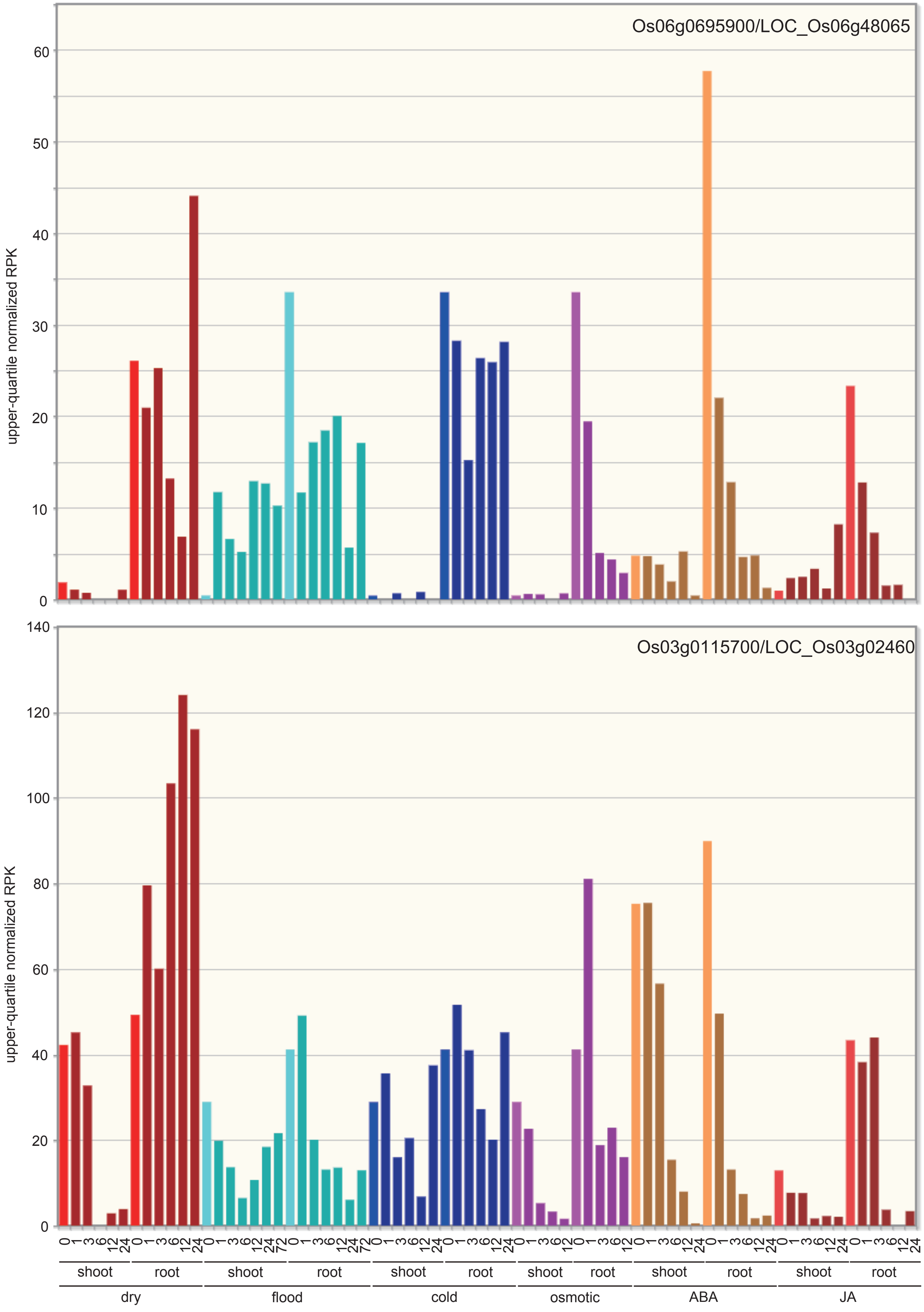
Os06g0695900/LOC_Os06g48065
Os03g0115700/LOC_Os03g02460
upper-quartile normalized RPK
shoot
root
dry
flood
cold
osmotic
ABA
JA

Supplemental Figure 5 The expression of awn genes, Os06g0695900/LOC_Os06g48065, Os03g0115700/LOC_Os03g02460

The expression of Os06g0695900/LOC_Os06g48065 (A and C) and Os03g0115700/LOC_Os03g02460 (B and D). Temporal and spatial expression was investigated in Rice X pro, while the expression under abiotic stress condition was searched in TENOR (Transcriptome ENcyclopedia Of Rice) database. DAF, days after flowering; ABA, abscisic acid; JA, jasmonic acid.
