# Supplementary material for: Low mutation rate of spontaneous mutants enables detection of causative genes by comparing whole genome sequences
Source: Front Plant Sci. 2024 Apr 4;15:1366413. doi: 10.3389/fpls.2024.1366413 (PMC11024370; doi:10.3389/fpls.2024.1366413)
Supplement: Supplementary file 6 [file DataSheet_6.pdf]

LOC_Os04g54230::32297556-32298132 Os04g0635000 "wound induced protein, putative, expressed"

```
LOC_Os04g54230.1        MASGGGKAKASSWAAAMSVGTVEALKDQAGLCRWNYAFRTLQQRGRQQAVAGT-SGAKSGGGA-R-ALQPAAAAAARRKA     77
Si011440m               MASGGGKAKASSWAAAMSVGTVEALKDQAGLCRWNYAFRSAQQRARGAVA-GTGSGSNAHALPSS-SSGGAAAAAAARRKA     78
Pavir.Gb00305.1.p       ----MAAAAKASWMVAMSVGAVEALKDQAGLCRWNYALRSVHRAAKANAPSF----------AQAKKLAPAA-ERRRAD-     64
GRMZM2G106413_P01       -MAAAAATKASSWMVAMSVGAVEALKDQGGLCRWNYALRSVYRTAKANVRSG----SRSFAAQRDKDLAPAA-EKGRQDK     74
Sobic.006G230700.1.p    MASGSGKAKATSWAAAMSVGTVEALKDQAGLCRWNYAFRQAAQQRARRPATAGASGSAGGAGSSA-SGCAASAAAARRKA     79
AT4G10270.1             -----MSSTSKAWTVAVSIGAVEALKDQLGLCRWNYILRSVNQHLRNNVRSV----SQGKRFSSSSVSAAVTSSGESEKA     71
Glyma.13G282400.1.p     -----MSAASRAWIVASSIGAVEALKDQLGVCRWNHALRSLQQHAKSNIRSY----TQAKTLSSATSAAV-----SNKVK     66
Solyc07g054780.1.1      -----MSSSRRAWIVAASVGAVEALKDQVGLCRWNYPLRSLAQHTKNNVRSY----SQAKKLSSSIT-------TKSEKM     64
                           :   :* .* *:*:******* *:****: :*   :

LOC_Os04g54230.1        QQQEEELRTVMYLSNWGPNN*---     97
Si011440m               RQQEEELRTVMYLSNWGPNN*---     98
Pavir.Gb00305.1.p       -KAEEGMRTVMYLSCWGPN*----     82
GRMZM2G106413_P01       AAAEEGLRTVMYLSCWGPN*----     93
Sobic.006G230700.1.p    KQQEEELRTVMYLSNWGPNN*---     99
AT4G10270.1             KKAEESLRTVMYLSCWGPN*----     90
Glyma.13G282400.1.p     RTKEESMRKVMDLSCWGPNTSRF*     89
Solyc07g054780.1.1      EKSEESLRKVMYLSCWGPN*----     83
                        ** :*.** ** ****
```

LOC_Os05g05920::2967576-2968672 Os05g0151100 "desiccation-related protein PCC13-62 precursor, putative, expressed"

```
LOC_Os05g05920.1        --------------------------------------------------------------------------------     0
Si024682m               --------------------------------------------------------------------------------     0
Pavir.J24637.1.p        --------------------------------------------------------------------------------     0
GRMZM2G327051_P01       --------------------------------------------------------------------------------     0
Sobic.009G046000.1.p    --------------------------------------------------------------------------------     0
AT3G62730.1             --------------------------------------------------------------------------------     0
Glyma.15G138000.1.p     --------------------------------------------------------------------------------     0
Solyc01g099580.1.1      MGKTEENNAFEHFSHGHQFVYINKQSNAICFGCRLNILPGKFYYKCESTCSFFLHQECFNMPKSLQHPVDPIHRLTLLTT     80
143620                  --------------------------------------------------------------------------------     0
Pp3c7_6750V3.1.p        --------------------------------------------------------------------------------     0

LOC_Os05g05920.1        --------------------------------------------------------------------------------     0
Si024682m               --------------------------------------------------------------------------------     0
```

```
Pavir.J24637.1.p          --------------------------------------------------------------------------------    0
GRMZM2G327051_P01         --------------------------------------------------------------------------------    0
Sobic.009G046000.1.p      --------------------------------------------------------------------------------    0
AT3G62730.1               --------------------------------------------------------------------------------    0
Glyma.15G138000.1.p       --------------------------------------------------------------------------------    0
Solyc01g099580.1.1        IPSSSKCNACRKEILGFSYACANCSTYYHTLCLLALPLSIEMSSHCHKLDLEFCPPYDFECDLCKKPSYKGWLYHCSSCE    160
143620                    --------------------------------------------------------------------------------    0
Pp3c7_6750V3.1.p          --------------------------------------------------------------------------------    0


LOC_Os05g05920.1          --------------------------------------------------------------------------------    0
Si024682m                 --------------------------------------------------------------------------------    0
Pavir.J24637.1.p          --------------------------------------------------------------------------------    0
GRMZM2G327051_P01         --------------------------------------------------------------------------------    0
Sobic.009G046000.1.p      --------------------------------------------------------------------------------    0
AT3G62730.1               --------------------------------------------------------------------------------    0
Glyma.15G138000.1.p       -------------------------------------------MH---------HHFGKK----------NLK----QNM    14
Solyc01g099580.1.1        FDAHISCAITHTDERKTEKCDELMELLSIYMKGTEETSVSQDQLHQYQAQQTPSYQFSDQCFSIDLTKSQQLNDEQTRSM    240
143620                    --------------------------------------------------------------------------------    0
Pp3c7_6750V3.1.p          --------------------------------------------------------------------------------    0


LOC_Os05g05920.1          -------------------------------MAARARRAVAVAVAVLHLLLLTASQL----AAPALSA-MAPPAAPLPGS    44
Si024682m                 --------------------------------MARARDAALLL---LASVLL-----------PLAAA-TIVPSAPAAAP    33
Pavir.J24637.1.p          --------------------------------MARARDATVLL---ASAVLL-----------PLAAA-TLLP---AAGP    30
GRMZM2G327051_P01         --------------------------------MARARDAA------LHLLLLAAGALLLLAVVSQATV-VP-PSVPAGAP    40
Sobic.009G046000.1.p      --------------------------------MARARDAAALH---RLLLVLAAGALLLLAVVSQATV-VPPPSVPTSAP    44
AT3G62730.1               --------------------------------------MFKL--------CLVLVL----VIA------VDAN-------    17
Glyma.15G138000.1.p       APQISRGRVPIVVLLA----------------------------------SLVLPL----LFQ--------E--------    40
Solyc01g099580.1.1        DT-KEKSNVAYVTLANEIGSEVWMGLGREMEKAYHTNDSNKM--------SFSFSY----AIN--------T--------    291
143620                    -----------------------------MGLIFGTSSVVVL--------ILLFSGFFV-SYA------HDEE----AC-    31
Pp3c7_6750V3.1.p          --------------------------------MMGTRNVL----------VFAVGLFLVLSFAAASGSKHDDNHELMIG-    37
                                                                          :


LOC_Os05g05920.1          ADPRCHASPPRRGAVAVYPSDMAHIQFLLNAKFVEAEWFLHGALGRGIDFIDGALSGGGPPPTGARKATLDFRATEVAAE    124
Si024682m                 EDPRCRATAPRRGALAVYPSDMEQLQFLLNAKFVEAEWFLHAALGRGVDFLDRNLSAGGPRPSGARKAALDFRTTEVAAE    113
Pavir.J24637.1.p          EDPRCRATAPRRGALAVYPSDMEQLQFLLNAKFVEAEWFLHAALGRGVDFLDRDLAAGGPRPAGARRAALDFRTTEVAAE    110
GRMZM2G327051_P01         EDPRCRALAPRRGALAVYPSDMEQLQFLLNAKFVEAEWFLHAALGRGVDFLDRNLSGGGPRPSGARKADLDFRTTEVAAE    120
Sobic.009G046000.1.p      EDPRCRATAPRRGALAVYPSDMEQLQFLLNAKFVEAEWFLHAALGRGVDFLDRNLSGGGPRPSGARKADLDFRTTEVAAE    124
AT3G62730.1               ---PAK-SGSCSCSGNISASDVDRVHFAMNLEFTEAEFFLKGATGKGLDAYNATLAKGGPPPIGAKKANLDPITNRIIEE    93
Glyma.15G138000.1.p       ---YSS-SSVFIASASASESDVDLLEFPLNLEYLEAEFFLFGSLGHGLDVVAPNLSEGGPPPIGARLARLENLIRDIILQ    116
Solyc01g099580.1.1        ---NCP-SGYPKKGVGVEKDDIDKMQFAVNLEFLEAEYFLWASYGFGLDVVAPNLPMSGPPPIGARKANLDQLTNNIIME    367
143620                    --GEVS-SAAFAPKGDVTPDDKKLLAFPLNLEYLETEFFAYGALGYGLDKLDPGLAENGPAPKGAQKAKLDILVRDIIAQ    108
```

```
Pp3c7_6750V3.1.p        --GLDI-DYKCRPGNYTTTKDKELTQVALNLEYFEAEYFLWGAYGYGLDKIAPYLVDGGPPPIGAQKANLDAYYTDIYIQ        114
                                     .*    . :* :: *:*:*  .: * *:*     *   ** * **: * *:     :  :

LOC_Os05g05920.1        LGYQEVGHIRAITQSM-----GGFPRPAIDLSDAVFAAVMDDA----MATRLDPPFDPYASSVNFLLASYILPHITASAA        195
Si024682m               LGYQEVGHIRAIRQAV-----GGFPRPAIDLGADRFAMVMDDA----MGVRLDPPFDPYASSVNFLLASYVFPHITAAAT        184
Pavir.J24637.1.p        LGYQEVGHIRAIRQAV-----GGFPRPAIDLGADRFAMVMDDA----MGARLDPPFDPYNSTVNFLLASYVFPHITAAAT        181
GRMZM2G327051_P01       LGYQEVGHIRAIRQAV-----GGFPRPPIDLGADRFAMVMDDA----MGVRLDPPFDPYAGPVNFLLASYVFPHVTAAAA        191
Sobic.009G046000.1.p    LGYQEVGHIRAIREAV-----GGFPRPPIDLAPDRFAMVMDDA----MGVRLDPPFDAYAAPVNFLLASYVFPHVTAAAA        195
AT3G62730.1             FGYQEIGHLRAITDMT-----GGIPRPLINLTRENFAVFMDRA----VGRKSNPRFDPYANSLNYLLASYYIPYVGLTGY        164
Glyma.15G138000.1.p     FGLQEVGHLRAIKSTV-----RGFPRPLLDLSTASFAKVMNSA----FGRPLVPPFDPYANSINYLLASYVIPYVGLTGY        187
Solyc01g099580.1.1      FANQEVGHLRSLNSTV-----GVFPRPLLDLSAKHFAKIFDDA----FGHKLVPPFDPYRDSLSYMLSCYVIPYVGLVGY        438
143620                  FALQEVGHLKAIKGVVK---EEGFPRPLLDLSVENWNTIMEKA----LGIKLDPPFSPYENSLNYMLASYAIPYVGLTGY        181
Pp3c7_6750V3.1.p        MGLQEVGHLRAIKRALGDPPRCAFPRTQLDISKKTWADTMDKAFLQTFGEKLNPPYDPYEDSLKYLISTYTIPYVGLTGY        194
                      :. **:**::::         :**  :::    :   :: *    ..    * :. *   :.:::: * :*::   .

LOC_Os05g05920.1        ----------------ASMLAVEAGQDAVIRMMLYERADEVVAPYKGRTVAEFTRRISEWRNAASR-CGAKDEGVKVLDR        258
Si024682m               MGISSSLMGFLSKRLQASILAVEAGQDAVIRLLLYQRADEAVPPYQGHTVADFTRRISEWRNRMSG-CGAKDEGVKVLDR        263
Pavir.J24637.1.p        MGISSSLMGFVSKRLQSSILAVEAGQDAVIRLLLYQRADEAVPPYQGHTVADFTRRISEWRNRMSG-CGAKDEGVKVLDR        260
GRMZM2G327051_P01       MGIGPSLMGYASKRLQASILAVEAGQDAVIRLLLYQRADDAVPPYQGHTVADFTRRISDWRNRMSG-CGAKDEGVKVLDR        270
Sobic.009G046000.1.p    MGIGPTLMGYASKRLQASILAVEAGQDAVIRLLLYQRADELVPPYQGHTVADFTRRISDWRNRMSG-CGDKDEGVKVLDR        274
AT3G62730.1             VGTIPYLVYFNIKKLVAGLLGVESGQDAVIRTLLYERQNEKVEEYGGVTVAELTNEISNLRNELGM-CGIKDEGLCV-PL        242
Glyma.15G138000.1.p     VGANPLLQNATSKRLVAGLLGVESGQDAVIRTLLYERQASLVQPYK-VTVAEFTDRISMLRNKLGN-AGVKDEGLVV-PR        264
Solyc01g099580.1.1      VGTNPNINGYETKRLLAGLLGVESGQDAVIRMYLYERAAELVSPYH-YTVADFTSRISGLRNKLGN-CGIKDEGVYV-QS        515
143620                  VGANPLTQSSDGKRLLAGLLGVESGQDAVIRTYLYERKDTVVEPYK-LTVHEITSKLSLLRSNLDDATGIDDEGLVV-PK        259
Pp3c7_6750V3.1.p        VGANPELKGYNAKKLVAGLLGVESGQDAIIRTEMYRQKNKKVSPYK-YTVADFSNAISNLRNNLSH--AFVDEGLVV-PN        270
                                   :.:*.**:****:**  :*.:    *  *   ** :::  :*  *.      .  ***: *

LOC_Os05g05920.1        RQGAERRTVSNILGAGDDSLGFARTPAEVLRILYGSGNEQVPGGFLPRGGNGTIAKGFFQLA*-------------  320
Si024682m               QQGAERRTISNILGAGDDSLGFQRTPAEVLRILYGSRNEQIPGGFLPRGANGTIARGFFQLA*-------------  325
Pavir.J24637.1.p        QQGAERRTISNILGAGEDSLGFQRTPAEVLRILYGSRNEQIPGGFLPRGANGTIARGFFQLA*-------------  322
GRMZM2G327051_P01       RQGAERRTISNILGAGEDSLGFQRTPAEVLRILYGSRNEQIPGGFLPRGANGTIARGFFQLA*-------------  332
Sobic.009G046000.1.p    KQGAERRTISNILGAGEDSLGFQRTPAEVLRILYGSRNEQIPGGFLPRGANGTIARGFFQLA*-------------  336
AT3G62730.1             WLGAENRTTSNILSADPYSLSYDRTAQEILRVMYGTGDEHRPGGFWPCGANGRIARMFLDEGCYGEYCVVCSHDN*  317
Glyma.15G138000.1.p     VQGAEGSVTDNILAGDKDSLSYPRTPEEILRIIYGGGDEHVPGGFYPNGACGRIAKSYLKYTT*------------  327
Solyc01g099580.1.1      PLGAENRTRSNVLSANFGSLSYKRTPAEILRIVYGSGDEHVPGGFYPNGANGKIAKEFLK*---------------  575
143620                  CLGAEQKIEGNILVGDKFSLSFARTPQQVLEIVYGTGDARKPGGFYPDGASGAIATKLRKAANVYF-*--------  325
Pp3c7_6750V3.1.p        ELGAEMMVTGNILSADNDSLSYPRTAEQVFETVYGTGDASKPGGFYPKGCQGVIAASYLD*---------------  330
                       ***    *:* .   **.: **  :::. :**  :   **** * *  * **    .
```

LOC_Os06g26234::15335687-15365531   Os06g0367100   "1,4-alpha-glucan-branching enzyme, putative, expressed"

```
LOC_Os06g26234.1        -MDSCAL----------------------------FLSTPRPPPL---VPAHRRRPLASR--------SGLRRREGC--      38
Si008087m               -MSSCPL----------------------------FLMTPRPPPLL---VHADRYPPI-GSAYLPTSRRPCPGRRHGY--    45
Pavir.Da01007.1.p       -MASCPL----------------------------FLMTPRPPPAL---VDASRFPPLAGSADLPTSRRPRPGRRHGH--    46
GRMZM2G005298_P01       -MASCPL----------------------------FLLVPRPPPSL---VGADRCPGSAGCSDLPTSCRPHPGRRHGY--    46
Sobic.003G213800.1.p    -MTSCPL----------------------------FLLVPRPPPPL---VGADGCPTSAGRAHLPPSCRPHPGRRHGY--    46
AT3G20440.2             -MVSLSN----------------------------QTRFSFHPNNL---VVSEKR--RLG---ISGVNF---PRKIKL--      38
Glyma.18G092600.1.p     MSSTLSI----------------------------PLGFCFPPTVA---TSFPHS--QTK---PQNVTF---QRRKTT--      39
Solyc07g064830.2.1      -MLSLSD----------------------------SLRISSPLSDS---RLSFLS--QTGSRTSCQFKFVRSRRARVS--    44
165481                  --------------------------------------------------------------------------------    0
Pp3c1_1810V3.1.p        MALSTGIQCVPAFKSNLVSVEAVTWASQASLSGVKFRGYERSGLGLRQSLGYLRDRHVVGGRVI-----RSIGRRASRTC    75


LOC_Os06g26234.1        -PCSCASSSSSSGRAGSQDRPPRPWQQKQRTQRP-G--RGEAIDPVGFLAKHGISDRAFAQFLRDRYKALKDRRWELHSR    114
Si008087m               -LFRCDSSSSSS---APRDRTPRPRQQRQRSQRP-GGRRVDAVDPVGFLAKLRVSDRAFAQFLRDRHKALKDRRWELCSR    120
Pavir.Da01007.1.p       ---RCDSSSSSS---APRDRPPRPRQQRQRSQRP-GGR-GDVVDPVGFLAKLGVSDRAFAQFLRDRHKALKDRRWELCSR    118
GRMZM2G005298_P01       -PFRCNSSSSSS---APRERPPR--------QRP-GG-RGDAIDPVGFLTKLGVSDRAFAQFLRDRHKALKDRRWELCSR    112
Sobic.003G213800.1.p    -PFRCDSSSSSP---APRERPPRSRQQRQ--QRP-GG-RGDAVDPVGFLAKLGVSDRAFAQFLRDRHKALKDRRWEVCSR    118
AT3G20440.2             -KITCFAAERPR----QE-KQK------KKSQSQSTSDAEAGVDPVGFLTRLGIADRIFAQFLRERHKALKDLKDEIFKR    106
Glyma.18G092600.1.p     -TKACAASENPN----QR-QNPKQNQKEAKTKNAGDDDGEKGINPAGFLAKRGISHKAFAQFLRERYKVLKDMKDEILKR    113
Solyc07g064830.2.1      -RCRCSATEGPT----PK-RRK------QIPEKYKQSEEEKGIDPVGFLSKYGITHKAFAQFLRERYKSLKDLKDEILTR    112
165481                  --------------------------------------------------------------------------------    0
Pp3c1_1810V3.1.p        KRIGCVRMAGAEEDVAQP--TKKGNTQSKNNVAEEGDTNRGGVNPVGFLKEKGLKTKAFQTFTRERYKALKDLKQLIAER    153


LOC_Os06g26234.1        LIDLKEASSGFELMGMHRHRQHRVDFMEWAPGARYCSVVGDFNQWSTTENCAREGHLGHDDFGYWTIILEDKLREGQEPD    194
Si008087m               FIDLKEVSSGFELLGMHRHRQHRMDFMEWAPGARYCSLVGDFNEWSPTENCAREGHLGHDDFGYWFIILEDKLREGQEPD    200
Pavir.Da01007.1.p       FIDLKEASSGFELLGMHRHRQHRIDFMEWAPGARYCSLVGDFNEWSPTENCAREGHLGHDDFGYWFIILEDKLRDGQEPD    198
GRMZM2G005298_P01       FIDLKEASSGFELLGMHRHRQHRIDFMEWAPGARYCSLIGDFNQWSPTENCAREGHLGHDDFGYWFIILEDKLREGQEQD    192
Sobic.003G213800.1.p    FIDLKEASSGFELLGMHRHRQHRIDFMEWAPGARYCSLVGDFNQWSPTENCAREGHLGHDDFGYWFIILEDKLREGQEQD    198
AT3G20440.2             HFDFRDFASGFELLGMHRHMEHRVDFMDWGPGSRYGAIIGDFNGWSPTENAAREGLFGHDDYGYWFIILEDKLREGEEPD    186
Glyma.18G092600.1.p     HENFMILASGFELLGMHRHPEHRVDYMEWAPGARYCAIIGDFNGWSPTEDCAREHYFGHDDFGYWFIILQDKLREGEEPD    193
Solyc07g064830.2.1      HFSLKEMSTGYELMGMHRNVQHRVDFLEWAPGARYCALIGDFNGWSTTRNCAREGHFGHDDYGYWFIILEDKLREGEEPD    192
165481                  MEIKRKFFHRYDILGMRHHFFHYVEYFEWAPGATSCSLIGDFNNWDCTKNRAEKGYFGRDDYGTWRITVEDKLREGQEKD    80
Pp3c1_1810V3.1.p        DEDLVEFANAYEDMGMHRNPGHHVEFYEWAPGARFCSVVGDFNNREHRKHFAREGYFGRDDFGYYHVRIDDVLREGEEED    233
                              :: :**::.  * ::: :*.**:   :::****  .  .. *.:  :*:**:* : : ::* **:*:* *


LOC_Os06g26234.1        EYYFQEYNYADDYDKGDNGVDVEELIHRMNEEYWEPGEIKSQKSRLE-VVAKLYEQMFGPNGP-----------------    256
Si008087m               EYFFQEYNYVDDYDKGDNGVDAEEIMHRMKEEYWEPGEIRSHKSQLE-MVVKLYEQMFGPNGP-----------------    262
Pavir.Da01007.1.p       EYFFQEYNYVDDYDKGDNGVDAEEIMHRMKEEYWEPGEIRSRKSQLE-MVVKLYEQMFGPNGP-----------------    260
GRMZM2G005298_P01       EYFFQEYNYVDDYDKGDNGIDADEIMRRMKEEYWEPGEIRSRQSQLE-MVVKLYEQMFGPNGP-----------------    254
```

```
Sobic.003G213800.1.p      EYFFQEYNYVDDYDKGDNGVDAEEIMSRMKEEYWEPGEIRSRQSQLE-MVVKLYEQMFGPNGP-----------------      260
AT3G20440.2              ELYFQQYNYVDDYDKGDSGVSAEEIFQKANDEYWEPGEDRFIKNRFE-VPAKLYEQMFGPNSP-----------------      248
Glyma.18G092600.1.p      KYYFQMYNYVDDYDKGDSGVSVEELIKKANEKYWQPGEDRFVNNRFE-GPVKLYEQIFGPNGP-----------------      255
Solyc07g064830.2.1       KLYFQQYNYADDYDKGDTGITIEEIFKKANDEYWEPGEDRFIKSRYE-VAAKLYEEMFGPNGS-----------------      254
165481                  P-HWQEYNYSVEYDRGDGDIDIEALYQKMEDEYWEPGEDQYLKDTRP-FEEALFKSIFGENFGCIKVGKEEEV-------      151
Pp3c1_1810V3.1.p         N-ATQEYNYDADYDKGDEDIDEDALFERIDQEYWDPGEDEFMSGHKDDLAQELFTTIFGKDLDPMEMVKDISAKYKSKKK      312
                          * ***  :**:**  :  : :  : .::**:*** .  .        *:  :** :

LOC_Os06g26234.1         -----------------------QTEEELGDIPDAETRYNEWKALQKD-DSASSLPCYDIIDN--GQEFDIFNVATDRVS      310
Si008087m               -----------------------QTEEELGEIPDAQTRYNEWKALQKT-DLSSMSPSYDIIDS--GQPFDIFNVVTDGAS      316
Pavir.Da01007.1.p        -----------------------QTEEELGEIPDAQTRYNEWKSLQKA-DSSSLSPSYDIIDN--GQPFDIFNVVTDRAS      314
GRMZM2G005298_P01        -----------------------QTEEELGEIPDAQTRYNEFKASQKA-DSS-SQPSYDIIDN--GQEFDIFSVVTDRAS      307
Sobic.003G213800.1.p      -----------------------QTEEELGEIPDAQTRYNEFKASEKA-DSLSMWPSYDIIDN--GQPFDIFNVVTDRAS      314
AT3G20440.2              -----------------------QTLEELGDIPDAETRYKQWKEEHKD-DPPSNLPPCDIIDKGQGKPYDIFNVVTSPEW      304
Glyma.18G092600.1.p      -----------------------QTIEDIPDIPDPETRYKAWAAEHGP-SPTAAI--------DSGKEYDIYNVIVDPQW      303
Solyc07g064830.2.1       -----------------------QTEEELEAMPDAATRYKTWKEQQKI-DPASNLPSYDVVDS--GKEYDIYNIIGDPES      308
165481                  NELAYN-----------------TEDEETMDYSKFPPGYEEWVKKTDQA----HLPPLK----QDPTRYSEPTVVDDPVW      206
Pp3c1_1810V3.1.p         KKMYYDDDDSGDDDDDDEYEREPQTLEEFKA--AVESHMEEWLASNAEAQKGKDLPSILV--EDDGINRDEMELVDDPVW      388
                                              *:          : :                          .   :  .

LOC_Os06g26234.1         FEKFQGKSPPLAYWVEMRKGRKAWLEKYVPAISHKDKYRVYFNTPDGGLERIPAWATYVLPDAEGKQSYAVHWDPPPEEI      390
Si008087m               FEKFQAKKPPLAYWVEMRKGRIAWLEKYVPTISHKDKYRVYFNTPDGALERVPAWATYVLPDAEGKQSYAVHWEPPPEEV      396
Pavir.Da01007.1.p        FEKFQAKKPPLAYWVEMRKGRKAWLEKYVPTISHKDKYRVYFNTPDGALERVPAWATYVLPDTEGKQSYAVHWEPPPEEI      394
GRMZM2G005298_P01        FEKFQAKKPPLAYWVEMRKGRIAWLEKYVPTISHKDKYRVYFNTPDGALERIPAWAAYVLPDAEGKQSYAVHWEPPPEEI      387
Sobic.003G213800.1.p      FEKFQAKKPPLAYWVEMRKGRIAWLEKYVPTISHKDKYRVYFNTPDGALERVPAWATYVLPDAEGKQSYAVHWEPPPEEI      394
AT3G20440.2              TKKFYEKEPPIPYWLETRKGRKAWLQKYIPAVPHGSKYRLYFNTPDGPLERVPAWATYVQPEDEGKQAYAIHWEPSPEAA      384
Glyma.18G092600.1.p      QEKIRALEPPVLYWFETRKGRKAWMKKYSPGIPHGSKYRVYFNTANGPLERVPAWATYVQPEVDGRQACAIHWEPSPEQA      383
Solyc07g064830.2.1       FKKFRMKQPPIAYWLETKKGRKGWLQKYMPALPHGSKYRVYFNTPNGPLERVPAWANFVIPDADGMQALAVHWEPPPEYA      388
165481                  RERVLAKKPPLPIWEYTVKGFKAWEKKYLPAIPHGSRVRVYFKTPEGPVERVPAWAKYVLPDPDGKMWSAVYWEPPIQER      286
Pp3c1_1810V3.1.p         AKRVEEKEWPENYWENFVKGRKAWEKKYIPGISHGDRYRAYLHTPEGPLERVPAWASYVLPDPDGNEVSAIFWDLPKDQQ      468
                        ::.   . *   *    **  .* :** * : * .: * *:.* :* :**:**** :* *: :*    *:.*:   :

LOC_Os06g26234.1         YKWRFERPKVKGSLRIYECHVGISGSEQKISSFQEFTSNVLPHIKDAGYNAIQLIGIVEHKDYSSVGYKVTNYFSVSSRF      470
Si008087m               YQWRFGRPKVKGSLRIYESHVGISGSEEKVSSFQEFTSKVLPHIKNAGYNAVQLIGVVEHKDYSSVGYKVTNYFAVSSRF      476
Pavir.Da01007.1.p        YKWRFGRPKVKGSLRIYESHVGISGSEQKVSSFQEFTSKVLPHIKNSGYNAVQLIGVVEHKDYSSVGYKVTNYFAVSSRF      474
GRMZM2G005298_P01        YKWRFGRPKVKGSLRIYECHVGISGSEQKVSSFQEFTSKVLPHIKKAGYNAVQLIGVVEHKDYSSIGYKVTNYFAVSSRF      467
Sobic.003G213800.1.p      YKWRFGRPKVKGSLRIYECHVGISGSEQKVSSFQEFTSKVLPHIKKAGYNAVQLIGVVEHKDYSSIGYKVTNYFAVSSRF      474
AT3G20440.2              YKWKYSKPKVPESLRIYECHVGISGSEPKVSTFEEFTKKVLPHVKRAGYNAIQLIGVPEHKDYFTVGYRVTNFFAASSRY      464
Glyma.18G092600.1.p      YKWKNMSPKVPKSLRIYEAHVGISGSEPKISSFNDFTDKVLPYIKEAGYNAIQLIGIVEHKDYFTVGYRVTNFFAVSSRY      463
Solyc07g064830.2.1       YKWKYKLPVKPKSLRIYECHVGISGQEPKISSFSDFISKVLPHVKEAGYNAIQIIGVVEHKDYFTVGYRVTNFYAVSSRY      468
165481                  HQWQHERPKPPKSLRIYECHVGMSSEEAGISTFKRFSQEVLPHVKKCGYNVVQLMGVQEHVDYSSVGYKMTNQFAVSSRF      366
Pp3c1_1810V3.1.p         YNWKFDRPSKPQTLRIYECHVGISGESPKIASFNDFTDTVLPRVAKAGYNVIQLFGIQEHADYSSVGYKVTNFFAISSRF      548
                       ::*:   *    :*****.***:*...  :::*. * . *** :  .***.:*::*: ** ** ::**::** :: ***:
```

```
LOC_Os06g26234.1          GSPDDFKKLVDEAHGLGLVVLLDIVHSYASADELVGLSLFDGSNDCYFHSGKRGHHKYWGTRMFKYDDIDVLHFLLSNLN   550
Si008087m                 GTPDDFKKLVDEAHGLGLVVLLDIVHSYASADELVGLSLFDGSNDCYFHSGKRGHHKYWGTRMFKYDDVDVLHFLLSNLN   556
Pavir.Da01007.1.p         GTPDDFKKLVDEAHGLGLVVLLDIVHSYASADELVGLSLFDGSNDCYFHSGKRGHHKYWGTRMFKYDDVDVLHFLLSNLN   554
GRMZM2G005298_P01         GTPDDFKKLVDEAHGLGLVVLLDIIHSYASADELVGLSLFDGSNDCYFHSGKRGHHKYWGTRMFKYDDVDVLHFLLSNLN   547
Sobic.003G213800.1.p      GTPDDFKKLVDEAHGLGLVVLLDIVHSYASSDELVGLSLYDGSNDCYFHSGKRGHHKYWGTRMFKYDDVDVLHFLLSNLN   554
AT3G20440.2               GTPDDFKRLVDEAHGLGLLVFLDIVHSYAAADQMVGLSLFDGSNDCYFHYGKRGHHKHWGTRMFKYGDLDVLHFLISNLN   544
Glyma.18G092600.1.p       GTPEDFKRLVDEAHGLGLLIILEIVHSYAAADEMVGLSMFDGSNDCFFRSGKRGQHKFWGTRMFKYGDPDVLHFLLSNLN   543
Solyc07g064830.2.1        GTPDDFKRLVDEAHGLGLLVFLEIVHSYAAADEMVGLSLFDGTNDCYFHTGKRGHHKFWGTRMFKYGDLDVLHFLLSNLN   548
165481                    GTPEDFKFLVDTAHGLGLLVFMDIVHSHVAPDEVCGLAMFDGANDCFLHY----------------GEHEVKRFLLSNLK   430
Pp3c1_1810V3.1.p          GTPEDFKRLVDTAHGLGLMVVMDIVHSHAAPNEGNGLASFDGANDCYFYPGRRGHHKRWGTRMFKYGEYEVLRFLLSNSK   628
                          *:*:*** *** ******::.::*:**:.: ::  **: :**:***::               : :* :**:** :

LOC_Os06g26234.1          WWVTEYRVDGFQFHSLPSMLYTHNGFSTFTGATEEYYNQYVDEDALIYLIIANEMLHELHPDIITIAEDATFYPGLCEPT   630
Si008087m                 WWVTEYRIDGFQFHSLSSMLYTHNGFSTFTGTMEEYCNQYVDKDAIIYLILANEMLHDLHPDIITIAEDATFYPGLCEPT   636
Pavir.Da01007.1.p         WWVTEYRIDGFQFHSLSSMLYTHNGFSTFTGAMEEYCNQYVDKDALIYLILANEMLHDLHPDIITIAEDATFYPGLCELT   634
GRMZM2G005298_P01         WWVTEYRIDGFQFHSLSSMLYTHNGFSTFTGAMEEYCNQYVDKDALIYLILANEMLHRLHPDIVTIAEDATFYPGLCEPI   627
Sobic.003G213800.1.p      WWVTEYRIDGFQFHSLSSMLYTHNGFSTFTGAMEEYCNQYVDKDALIYLILANEMLHQLHPDIITIAEDATFYPGLCEPT   634
AT3G20440.2               WWITEYQVDGYQFHSLASMIYTHNGFASFNNDLDDYCNQYVDRDALMYLILANEILHVQHPNIITIAEDATYYPGLCEPV   624
Glyma.18G092600.1.p       WWIVEYQIDGFQFHSVSSMMYTHNGFASFTGELEEYCNQYVDKDALVYLILANEILHSLHPNIITIAEDATFYPGLCEPT   623
Solyc07g064830.2.1        WWVEEYHVDGFHFHSLSSMLYTHSGFASFTGDMDEYCNQYVDKEALLYLILANEVLHALHPNVITIAEDATLYPGLCDPT   628
165481                    WWVEEYRIDGFYFHSVGSMLYTHNGFANFTGSLDEYCNQYVDMDAHIYLILANELLHNLTPRIITIAEDATLFPGLCASH   510
Pp3c1_1810V3.1.p          WWFMEYKVDGFYFHSVTSMLYTHNGFTPFTSGLDDYCNQYVDKDALIYLSLANEMLHQLSPNMITIAEDATFYPGLVDSI   708
                          **. **::**: ***: **:***.**: *.   ::* ***** :* :** :***:**   * ::******* :***

LOC_Os06g26234.1          TQGGLGFDYWVNLSIPEMWLWHLENVPEQEWSMNKIMRVLVNNN---SNMLSYVENHNQSISGRKSFAEIILYE-GKCSN   706
Si008087m                 TQGGLGFDYCVNLSVPEMWLWHLENVPEREWSMNKIMKVLVSSD---QKMLSYVENHNQ---------------------   692
Pavir.Da01007.1.p         TQGGLGFDYCVNLSVPEMWLWHLENVPEQEWSMSKIMKVLVSRN---QNMLSYVENHNQVIIVSLILFWFLNREKLLCNR   711
GRMZM2G005298_P01         TQGGLGFNYWVNLSIPEMWLWHLENVPEQEWSMNKIIKVLVSSN---QNMLSYVENHNQSISGRKSFAEII-LNSRECSV   703
Sobic.003G213800.1.p      TQGGLGFDYWVNLSVPEMWLWHLENVPEREWSMNKIIKVLVSSN---QNMLSYAENHNQSISGRKSFAEII-LNSGECSV   710
AT3G20440.2               SQGGLGFDYYVNLSASEMWVSLLDNVPDNEWSMSKIVSTLVANKEYADKMLSYAENHNQSISGGRSFAEILFGGVDNGS-   703
Glyma.18G092600.1.p       SQGGLGFDYYVNLSVPDMWSTFLESVPDHEWSMTKIVNTLVSNREHADKMLMYAENHNQSISGRRSFAEILFGEIDENS-   702
Solyc07g064830.2.1        SQGGLGFDYFTNLSASEMWLALLENTPDHEWCMSKIVSTLVGDRQNTDKMLLYAENHNQSISGGRSFAEILIGNSLGKS-   707
165481                    EQGGFGFDYYVSTAPSDMWLYLIEKVPLEEWSVKQIAESLLKLSESDGKALVYVENHSQSISGGKSLFQALIEKNVEYP-   589
Pp3c1_1810V3.1.p          NKGGLGFDYYVNSAPSEMWPFLIENVPIQEWSVTEITGTLITT-ENTTKALVYSENHNQSIVGGQSLAEALLGTSKESS-   786
                           :**:**:* .. :  :**   ::..* .**.:.:*   *:      : * * ***.*

LOC_Os06g26234.1          SSVDNDLIFRASSLLNIIKLITFTTSGGAYLNFIGNEFAHPKRIEFPMSSNDYSFCLANRQWELLD-KGVHKHIFNFDKD   785
Si008087m                 --------------------------------------------------------------------------------   692
Pavir.Da01007.1.p         GNQ-TYYF*-----------------------------------------------------------------------   718
GRMZM2G005298_P01         GSV-DDNLIRASSLLKLMKLITFTTSGGAYLNFMGNEFAHPEKVEFPMPSNDYSFELANRQWELLD-KVFHKHIFNFDKD   781
Sobic.003G213800.1.p      GSV-DDNLIRASSLLKIIKLITFTTSGGAYLNFMGNEFAHPERVEFPMPSNDYSFQFANRQWELLD-KGFHKHVFNFDKD   788
AT3G20440.2               -PGGKELLDRGISLHKMIRLITFTSGGRAYLNFMGNEFGHPERVEFPTQSNNFSFSLANRRWDLLE-SGVHHHLFSFDKE   781
```

```
Glyma.18G092600.1.p      -NHYKESLLRGSSLHKIIRLITLTIGGRAYLNFMGNEFGHPKRVEFPTSSNNNSYLLANRQWDLLTKDGVHRDLFAFDKD      781
Solyc07g064830.2.1       -SISQESLLRGCSLHKMIRLITSTIGGHAYLNFMGNEFGHPKRVEFPMSSNNFSFSLANRRWDLLE-DDVHYRLFSFDKD      785
165481                   ---DAVNMLKSVSMIKMIKLLTASLGGSAYLTFMGNEFGHTERVEFPRATNNFSYEFARRRWSLLD-DKWHAKLAEFDNA      665
Pp3c1_1810V3.1.p         ---KNISKLEGISLHKIIRLITLSLAGSAYLNFMGNEFGHPKWVEFPRAKNNNSFAHAYRRWDLLEEQGPHSQLAAFDQA      863


LOC_Os06g26234.1         IMSLDGKERLISGGSPI-VHHCDDTSMIIYFTRGPFLFVFNFNPDASYQLYSVGVDEAGEYQLILNTDETKYGGRGELTS      864
Si008087m                --------------------------------------------------------------------------------      692
Pavir.Da01007.1.p        --------------------------------------------------------------------------------      718
GRMZM2G005298_P01        VMSLDENERIISRGPPN-ILHCDDTSMVISFTRGPLFFIFNFNPQVSHQSYRVGVDEAGEYQLIFNTDETKYGGCETLKS      860
Sobic.003G213800.1.p     VMSLDENERIISRGSPN-ILHCDDTSMVISFTRGPFLFVFNFNPEVSHQSYHVGVEEAGEYQLIFNTDETKYGGCGTLKS      867
AT3G20440.2              LMDLDKSKGILSRGLPS-IHHVNDANMVISFSRGPFLFIFNFHPSNSYEKYDVGVEEAGEYTMILNSDEVKYGGQGIVTE      860
Glyma.18G092600.1.p      MMKLDENVKVLSRNIPN-IHHVNDSSMVISYIRGPLLFIFNFHPKDSYDSYSIGVEEAGEYQIILNTDEIKYGGQGILKE      860
Solyc07g064830.2.1       MMDLDKNGRILSRGLAN-IHHVNDTTMVISYLRGPNLFVFNFHPVNSYERYIIGVEEAGEYQVTLNTDEKKYGGRALLGH      864
165481                   LMAIEQKYLFLNSNAPATNLQVDDSSKTVVFTRDNLIFAYNFHPRKSADEYEILVDEPGQYELLLDTDDVKYGGMGRLKT      745
Pp3c1_1810V3.1.p         LMEVDETHNILGQGLPN-MCHVNDTTKVIVYTRGNLLFAFNFHVTDTYEMYKVGVQVAGEYELVLNSDQPNFGGLGQLQE      942


LOC_Os06g26234.1         NQYMKRTSDNRVGGCRNSLELTLPSRSAQVFKLVRILRI*-----      903
Si008087m                ---------------------------------------------      692
Pavir.Da01007.1.p        ---------------------------------------------      718
GRMZM2G005298_P01        SQYMRRTSDKRADGCRNSLELALPSRSAQIYKLVRILRI*-----      899
Sobic.003G213800.1.p     SQYMRRTCDKRADGCRNSLELALPSRSAQVYKLVRILRI*-----      906
AT3G20440.2              DHYLQRSISKRIDGQRNCLEVFLPSRTAQVYKLTRILRI*-----      899
Glyma.18G092600.1.p      EQYFLKTISRRVDGLRNCLEVSLPSRTSQVYKLRRILRI*-----      899
Solyc07g064830.2.1       DQNIQRTISRRADGMRFCLEVPLPSRSAQVYKLTRILRA*-----      903
165481                   KQ-------KRIDVFSMKLSLTLPQLSAQVYRLAKIWDAAVSVM*      782
Pp3c1_1810V3.1.p         ADKLLNTTRRQSDGLPNTLLLVLPQLSAQVYKLARVFETSS*---      983
```

LOC_Os09g34070::20109768-20114569   Os09g0516300   "RNA recognition motif containing protein, expressed"

```
LOC_Os09g34070.1         -----------MSSEPP----PPQPQPQE----------AAGREASSSLSPAKESAAGGGGVGGSGAPETNTLWVGNLPA      55
Si028870m                -----------MSSEPP----PAGSPEAR----------A-------SASPPKDA-VASGAGAAAGFLETNTLWVGNLPS      47
Pavir.J04458.1.p         -----------MSSEPP----PADSPEAP----------A-------SASPPKDA-VASGGGAAAGVLETNTLWVGNLPA      47
GRMZM2G070038_P01        -----------MSSEPL----PAESPEAP----------AS-----ASGSPSKDA-VGTEVGAAAGSPETNTLWVGNLPS      49
Sobic.002G264200.1.p     -----------MSSESP----PTESPDAP----------ASGS-RSGSGSPSKDA-VGTEGGAAAGGPETNTLWVGNLPL      53
```

```
AT2G43410.2               --------------------------------------------MALSMKPF--------RA-DDSGFQSNNLWVGSLTP   27
Glyma.11G126400.1.p       --------------------------------------------MPLPAKPM--------RDFDESAPPSNNLWVGNLAA 28
Solyc06g053320.2.1        -------------------------------------------------------------MAPPGEIPSNNLWVGNIAP 19
413458                    -------------------------------------------------------------------------------- 0
Pp3c7_3950V3.1.p          MQLRTGTGNGEMATRRPAGLPPVGAPPLRGGFPVDYGPRVDDRRQGFSS--STSRPAHNGRDDLEDTPPSRHLWIGNVSQ 78


LOC_Os09g34070.1          QAAEDDVMASFSPHGALDCVMARAGPRSYAFVLFRSVPEARAALDALQGSKVKGSVVRLEFARPA--------------- 120
Si028870m                 YVSEGDLMALFAPHGALDCALARAGSRSYAFVLFRTPAEARAAVEATRGEKVKGAAMRTEFARPA--------------- 112
Pavir.J04458.1.p          HVSEGDLMALFAPHGALDCALARAGSRSYAFILFRTPAEARAAVEATRGEKVKGAAMRTEFARPA--------------- 112
GRMZM2G070038_P01         HVTEGDLLALFGPHGALDCALARAGSRSYAFVLFRSPAEAREAVEATRGEKVKGAAMRTEFARPVFVVADENATRTVLVE 129
Sobic.002G264200.1.p      HVTEGDLLALFGPHGALDCALARAGSRSYAFLLFRSPAEARAAVEATRGEKVKGAAMRTEFARPA--------------- 118
AT2G43410.2               ETTESDLTELFGRYGDIDRITV-YSSRGFAFIYYRHVEEAVAAKEALQGANLNGSQIKIEYARPA--------------- 91
Glyma.11G126400.1.p       DVTDADLMELFAKYGALDSVTS-YSARSYAFVFFKRVEDAKAAKNALQGTSLRGSSLKIEFARPA--------------- 92
Solyc06g053320.2.1        DVTDADLTSLFQKYGQLDSVTA-YSSRGFGFLYFKNINDSKEAKDALQGSLFHGNPLRIEFAKPA--------------- 83
413458                    -------------------------MRRFGVMFLVLLALAAVATELLKL----PNALRLENSLTLLLASRKS--LSLFAF 49
Pp3c7_3950V3.1.p          DASEAAIRDKFSQFGDVDSVTV-YSSRNYAFVNFRNLEDAVEAKTHLQGFVLGGMAIRIEYAKGA--------------- 142
                                             * :..:      :  *   :       :: * :


LOC_Os09g34070.1          ------------------------------RAVKNLWVGGISSSISKEELEEEFKKFGKVDGIAFSRDQTSAYIDFDKLE 170
Si028870m                 ------------------------------RAVRNLWVGGISPSVSKEELEEEFQKFGKVEGVAFSQDQTSAYVDFEKLE 162
Pavir.J04458.1.p          ------------------------------RAVRNLWVGGISPSVSKEELEEEFQKFGKVEGVAFSNDQTSAYVDFEKLE 162
GRMZM2G070038_P01         DTGRMPCLSIVNLADQA-------------RAVRNLWVGGISPSISKEELEGEFLKFGKVEGVAFSQDQTSAYIDFEKLE 196
Sobic.002G264200.1.p      ------------------------------KAVRNLWVGSISPSVSKEELEEEFQKFGKVEGVAFSQDQTSAYIDFEKLE 168
AT2G43410.2               ------------------------------KPCKSLWVGGIGPNVSKDDLEEEFSKFGKIEDFRFLRERKTAFIDYYEMD 141
Glyma.11G126400.1.p       ------------------------------KACKQLWVGGISQAVTKEDLEAEFHKFGTIEDFKFFRDRNTACVEFFNLE 142
Solyc06g053320.2.1        ------------------------------KPCKSLWVAGISKSVSKEELEDQFKGFGTIQEYKFIRDRNTAYIDFARLE 133
413458                    TDGTVEILLAIKRKDVKSSLLIRWFEECFTPECKQWSFQGVLLG------HDDFITNGVLLSLI---IRCCSELDNKSFP 120
Pp3c7_3950V3.1.p          ------------------------------TQSRHLWVGGISSNVTKEQIEGEFRKYGVLEDFKLLRERNCAFVDYVRME 192
                                                   :   . .:       . :*   * :       :  : ::  :


LOC_Os09g34070.1          DAISAH-----RALNGRVLGGQ--ELCVDFQRSRG----RAEWLETGS-----FNGR--TGP------------------ 214
Si028870m                 DAISAH-----RSLNGRTLGGK--ELCVDFQRSKG----RAEWSEASG-----FNGR--VSGPPADKRGTG--------- 215
Pavir.J04458.1.p          DAISAH-----RSLNGRTLGGK--ELCVDFQRSKG----RAEWSEASS-----FNGR--VSGLPGDKRATA--------- 215
GRMZM2G070038_P01         DAISAH-----RSLNGKMLGGK--ELCVDFQRSKG----RAEWSEASS-----FNGR--VPGPVGDKRGSG--------- 249
Sobic.002G264200.1.p      DAISAH-----RSLNGKTLGGK--ELCVDFQRSKG----RAEWSDASS-----FNGR--VSGPVGDKRGSG--------- 221
AT2G43410.2               DALQA------KSMNGKPMGGS--FLRVDFLRSQA--PKKEQWAGSYDNRNGNMNHK-------PQY--P---------- 192
Glyma.11G126400.1.p       DACQAM-----KIMNGKRIGGE--HIRVDFLRSQS--TKRDQLLDYGQ-----FQGK--NLGPTDAY-------S----- 194
Solyc06g053320.2.1        DAAEAL-----KNMNGKKFGGE--QIRVDYLRSQP--TRREQGPEYREMRDGQYHNR--NVGHPDSRLMPQDFARNYSDP 202
413458                    TQFRSVSLSFALCFDS-RSKGEVLELLIGFGEQLERFAHLGLWKAYAV-------GE--SRPSPELQLGNLDQVANYCWS 190
Pp3c7_3950V3.1.p          DAVSAV-----EALNRKRIGEE--ELRVDYGRSQP--SKRDSRGEQRGSQDGYSTQHGSQGGAG---------------- 247
                           :       ::     .  : : : ..                   .
```

```
LOC_Os09g34070.1         ----------AKGYGVRNRESNPTNVLWVGFPNT-----------AK--INEEALRQAMAVHGAVTNTKVFPTRQYAFVE   271
Si028870m                --------PPKGSAGSRMRDAQPTNVLWVAFPAS-----------YK-VIDEEMLRQAMSAFGVVTKIKIFQTRQYAFVE   275
Pavir.J04458.1.p         --------PLKGSAGTRTREAQPTNVLWVGFPGS-----------YK-VIDEEALKQAMSAFGVVTKIKIFQSRQYAFVE   275
GRMZM2G070038_P01        --------PLKSSAGVRMREAQPTNVLWVGFPGS-----------YR-VIDEEALKHAMSVFGVVTKIKVFQTRQYAFVE   309
Sobic.002G264200.1.p     --------PPKGSAGIRMREAQPTNVLWVGFPGS-----------YK-AISEDTLKQAMSAFGVVTNIKIFQTRQYAFVE   281
AT2G43410.2              --------H---SYEDFKGDVQPSKVLWIGFPPT-----------ATQCNDEQILHNAMILFGEIERVKSYPSRNFALVE   250
Glyma.11G126400.1.p      ---GQKRPLHSQPPMGRKGDSQPSNILWIGYPPA-----------VQ--IDEQMLHNAMILFGEIERIKSFPSRNYSIVE   258
Solyc06g053320.2.1       MHAGFRRQHPFQLP-VGQGHGQPSKILSIGYPPS-----------VH--VDEDMLHNAMILFGEINGIRTFYDRNFSLVE   268
413458                   MQHD-------RRCLKWQGRSWPPRLIRGAFDIIELALNRDRLVSTSQCYDST-LKLVKIHESTFELLRDIMNLLFAFAI   262
Pp3c7_3950V3.1.p         --------NGVEGRVKADKDGGPSEILWVGFPLP-----------SK--VDEDGLRRAFMPYGEVERVKTFPGRTYAFVQ   306
                                         * .::  .:                .. *: .    . .  :     :::.

LOC_Os09g34070.1         FATVGEASNA------KKNLDGRLFNDQRIQILFSNSELAPNKLDNPTAVSGFP----KS-EMYYDD-------------   327
Si028870m                FSSVVEAYNA------KTNLDGHLFNDPRIQILFSNSELAPNKLDNPTSLSGFP----RS-EMYSSD-------------   331
Pavir.J04458.1.p         FASVVEAYNA------KTNLDGHLFDDPRIQILFSNSELAPNKLDNTTSVSGFP----RSSEMYSSD-------------   332
GRMZM2G070038_P01        FANVAEACNA------KMNLDGHLFNDPRIQILFSNSGLAPNKLDNPTSVAGFP----SS-EIYSSD-------------   365
Sobic.002G264200.1.p     FANVAEAYNA------KMNLDGHLFNDPRIQILFSNSELAPNKLDNPTSVAGFP----RS-EMYSSD-------------   337
AT2G43410.2              FRSAEEARQC------KEGLQGRLFNNPRIKIMYSNDELPPEQDDTSFYSGMKR----SRTDMFNNDPSFVSSPH-----   315
Glyma.11G126400.1.p      FRSVDEARRA------KEGLQGRLFNDPRITIMYSISDLVPGSDYPGFFPGSNG----PKPDVLLNDHPFRPLQMDAFGH   328
Solyc06g053320.2.1       FRSVEEAQRA------KEGLQGKLFNDPRITIEYYSSGPAPGREYH---PSIIG----PTTDSYPNENSFQPAQMGMFGH   335
413458                   YPGSDETSQQFLQVCSNRGVDQQLRM------------------------------------------------------   288
Pp3c7_3950V3.1.p         FQKVEEATRA------KNALDGKLFDDPRVHIRYSKSEIGPIDSPRDGPPSRMVDRQGFSTEAIGGSRGIPSAGSDRFG-   379
                      :   *: .      :  :: :*

LOC_Os09g34070.1         ----------------GQYGA---------SDYFDPRRGRSRYFE--------------------------------YSG   350
Si028870m                ----------------GRH--GLGSGT--LQGYDPPRGGRSRHFD--------------------------------YGG   359
Pavir.J04458.1.p         ----------------GRH--GLGSGT--LQGYDPPRGGRSRHLD--------------------------------YGG   360
GRMZM2G070038_P01        ----------------GRLGPGIGSGT--LQGYDPPRGGRTRYYD--------------------------------YAG   395
Sobic.002G264200.1.p     ----------------SRQGPGVGSGT--LQGYDPPRGGRSRYYD--------------------------------YGG   367
AT2G43410.2              --------------STGIPGSM---RP--LRGTNERSYNGAEYNDVV--------------GKEPNWRRPSANGT-GILP   361
Glyma.11G126400.1.p      NRPMGPNNFPGQLPPSGIMGPNIPMRPFGPHSGVESVISGPEFNEINALHKF-QDGSSKSS-MGPNWKRPS---------   397
Solyc06g053320.2.1       NRPMLASNVPGHLPPFGIHGPEIPARPLGMQGRFDPTISGPEYTDLPVASKL-RDTSPHNVVGGPNWKAA----------   404
413458                   ------------------------------------VACTVYLYLSLMLKMVRRELLCDL--------------------   312
Pp3c7_3950V3.1.p         -----------------SPGRASANSNARLGGG-----LRPEYRPNALMAGLVDRGSSREPDIGPGMGRASHRNVSHVDD   437
                                                         .

LOC_Os09g34070.1         VPVSGGILPSPESGNPLLTG--RSA----QSTFDPREAKRLRLDAAADPYDT-------RAGSEGLYSAGY---------   408
Si028870m                LPTPGGILPPPE-------------------SFDPREAKRMRLDAGADPYEV-------RAGSTSLYSAGI---------   404
Pavir.J04458.1.p         LPTPGGILPPPE-------------------PFDPREAKRMRLDAGVDPYDV-------RAGSTSLYSTGL---------   405
GRMZM2G070038_P01        MPTPGGILSQPE-------------------PFDPREAKRMRLAAGADPYDV-------RAGSTGLYSAGY---------   440
Sobic.002G264200.1.p     VPTPGGILSQPE-------------------PFDPREAKRVRLDAGADPYDV-------RAGSAGLYSAGY---------   412
AT2G43410.2              SPTGPGILPSPAQGTRRPMRSNPDSWEGYDPAQLVRESKRTRRDGSVDGF-----------TPMGVDERS----------   420
Glyma.11G126400.1.p      -PPAPGMLSSPAPGARLPTRSTSGAWDVLDINHIPRDSKRSRIDGPLPVDEG-------PFPLRNIDDRGL---------   460
```

```
Solyc06g053320.2.1       -SPTPGMLSSPSGVQKAPSRSAIPGRDVFDSSQLQRESKRSRIDGAYDN----------SYPHKRTSD------------     461
413458                  ---------------------------------SLRDSLRRNVL-----VRRSLNAWFKELNPQSMEVKVDRGASITRRS    354
Pp3c7_3950V3.1.p         MEYSRGI-------RPDSRSSYDDAWDLPDADIVPRDSKRLRVYSGGGADSPGFDPWYEQRPQQSSDSGAYMGT------     504
                                                      *:: * .

LOC_Os09g34070.1         -----------------------S-----QRES--ARSERSSSPA-------------IRIHG--TVH------------     431
Si028870m               -----------------------R-----HRDSS-VHAEGSSSPA-------------IRVRG--TVH------------     428
Pavir.J04458.1.p         -----------------------R-----NRDSS-VHAEGSSTPA-------------IRVRG--TVH------------     429
GRMZM2G070038_P01        -----------------------R-----HQGSS-VHAEGSSSPV-------------IRVRG--TVH------------     464
Sobic.002G264200.1.p     -----------------------R-----QQGSS-VHAEGSSTPV-------------IRVRG--TVH------------    436
AT2G43410.2              ----------------------------FGRG-----------------------SVAARP-----IR------------     432
Glyma.11G126400.1.p      -----------------------ALEQTYGIDP--AIDGGGSGPYVNIQGKSHLGPVSSRITAGVHGV------------    503
Solyc06g053320.2.1       ------------------------RAEQYGLGP--FGTNVPSGPVTVGQANNSVSPLDARISPG---Q------------     500
413458                  AVPYLEHALLATKDPKHKQVQLVQIIAEYRRRLARKLAESKKEQ-----DTVYATP----IKGDDQIL----------IE     415
Pp3c7_3950V3.1.p         -------GVSSTYEPLR-----LPATPDYNLGLGGRSRAGLSGPESNI-----AMPIGTR--SGVGGHVPPPNSLVLPIS    565


LOC_Os09g34070.1         ----------RTSYLEHFWRGSIAKGGSP--VCRARCLPIRKG--VEIPLPDVVNCSARTGLDML-------AKHYRDAS     490
Si028870m               ----------RTSYLEHFWRGSISKGGSP--VCHARCLPITKG--SDIPLPDVINCSARTGLDML-------AKHYADAT     487
Pavir.J04458.1.p         ----------RTSYLEHFWRGNIAKGGSP--VCRARCLPITKG--SDIPLPDVINCSARTGLDML-------AKHYADAT     488
GRMZM2G070038_P01        ----------RTSYIEHCWRGSIAKGGSP--VCRARCLPITKG--SDIPLPDVMNCSARTGLDML-------AKHYADAT     523
Sobic.002G264200.1.p     ----------RTSYIDHCWRGSIAKGGSP--VCRARCLPITKG--SDIPLPDVMNCSARTGLDML-------AKHYADAT    495
AT2G43410.2              ----------GPPDSDHIWRGMIAKGGTP--VCCARCVPMGKG--IETKLPEVVNCSARTDLNML-------AKHYAVAI     491
Glyma.11G126400.1.p      ----------AQPDIDHIWRGVIAKGGTP--VCRARCVPIGKG--IGTELPDVVDCSARTGLDIL-------TKHYADAI    562
Solyc06g053320.2.1       ----------RLPGHNYIWHGTIAKGGTP--VCHARCVPIGES--IEFEIPEVVNCSARTGLDML-------TKHYADAV     559
413458                  TAQEHRLQEPELCNLDGLWLSPPPYEGNESVIFSVSCRDSSSHAFLILLLESITKVTERIDIRADAEKLRFESNLVFVPP     495
Pp3c7_3950V3.1.p         STYGKHNVEDVKGPEGWQWHGTIAKGGTP--VCRARCLPVGKG--IDATVPDVVNCTARTDLDML-------AKHVYQAG    634
                                          * .    *.   :  . *    .      : .: . : *  :         ::

LOC_Os09g34070.1         GFDIVFFLPDSEDDFVSYTEFLRYLGSKSRAGVVK---------VDGGTTLFLVPPSDFLRNV-LQVD-GPERLYGVVLH    559
Si028870m               GFDVVFFLPDSEDDFVSYTEFLRYLGSKSRAGVVK---------VDAGTTLFLVPPSDFLTNV-LQVD-GPERLYGVVLH   556
Pavir.J04458.1.p         GFDVVFFLPDNEDDFISYTEFLRYLGSKSRAGVVK---------VDAGTTLFLVPPSDFLTNV-LQVD-GPERLYGVVLH    557
GRMZM2G070038_P01        GFDIVFFLPDSEDDFVSYTEFLRYLGSKSRAGVVK---------VDAGTTLFLVPPSDFLTNV-LQVD-GPERLYGVVLH    592
Sobic.002G264200.1.p     GFDIVFFLPDSEDDFVSYTEFLRYLGSKSRAGVVK---------VDAATTLFLVPPSDFLTNV-LQVD-GPERLYGVVLH   564
AT2G43410.2              GCEIVFFVPDREEDFASYTEFLRYLSSKDRAGVAK---------LDDGTTLFLVPPSDFLTDV-LQVT-RQERLYGVVLK    560
Glyma.11G126400.1.p      GFDIVFFLPDSEDDFASYTEFLRYLSAKNRAGVAK---------FVDNTTLFLVPPSDFLTRV-LKVT-GPERLYGVVLK   631
Solyc06g053320.2.1       GFNVVYFLPNSEKDFASYTEFLGYLGSKDRAGVAK---------FANGTTLFLVPPSDFLTKV-LKVV-GPKRLYGVVLK    628
413458                  GERIGRFARDRECKKAHVQQFMATEEALNEAAEAKAASDKLIKSLSGGTELDYVQNEGGLRQLECEVWAKERQLAGVKAS     575
Pp3c7_3950V3.1.p         GFGVVFFVPEGDPDVPPYQDFMHYLGEKHRAGVAK---------LADGTTLFLVPPSEFSEKV-LKVP-GDNCLFGVVLK    703
                        *  :  *  : : .    :*:      .*. .*        .  * *  *  .    :  :*    . * **

LOC_Os09g34070.1         IPQMSAAAPASAPTPAVQRPQLTAPES---QPYYDEREI----P--LQRRYSMITPS-------NNHHRD----ADHR--    617
Si028870m               IPQMSAA-A-------ALRPQLTGPEL---QPYYDEREA----LPTSQRKYSIISPS-------DNGYSD----ADYR--    608
```

```
Pavir.J04458.1.p            IPQMSAA-A-------ALRPQLTGPEL---QPYYDERET----LPTSQRKYSIISPS-------DNGHHD----ADYR--           609
GRMZM2G070038_P01           IPQISAA-A-------ALRPQLTGTEQ---QPYYDERGT----LPTSQRKYSIISPN-------DSGHLD----ADYR--           644
Sobic.002G264200.1.p        IPQISAAAA-------ALRPQLTGTEQ---QPYYDERET----LPT-QRKYSIISPN-------GSGHLD----ADYR--           616
AT2G43410.2                 LPPPAVPVTASYR----------QESQ---SNPLHYMDQ----ARDS--------------------------------           590
Glyma.11G126400.1.p         FPPVPSSAPMQQP----------SHLR---VPTTQYMQQ----IPPSQTEYGLIPVK-------EEHILP----MDYN--           681
Solyc06g053320.2.1          FAHHMPSGTS-LP----------QESS---QPQYVDAPR----MPSSQAAYDAMPSV--------ERVPQ----MNYN--           676
413458                      VATLTSEVQRSKK-LCEERKQAEESLRRKRIEEFDARRR------ELESVYTALIRANMEAAAAREQHPAA--AFEYSA-           645
Pp3c7_3950V3.1.p            FQQPGPAPVVNYNPPAQQQQQVPSLGQ---HPPYSQQQLPSQHTPISQSQYPQNQAP-------PQHVPGIQEPAPYQGF           773
                           .

LOC_Os09g34070.1            -GSLREDSLHQLGQIL--------ARPRVDEGQ--VVQPNLAGIPTN--AGLQVQPSLQPDMIATLAKLLPSGQSSALVT           684
Si028870m                   -GSLREESMHHLGQLS--------GRPRVDEGQ--AVQPALAGFPTNQTAAAQVQPSVKPDIMATLAKLMPSVQSSPLVS           677
Pavir.J04458.1.p            -GSLREESMHHLGQIS--------GRPRMDEGQ--AVQPALAGFPTNQTAATQVQPPVKPDIMATLAKLMPSVQSSTLVS           678
GRMZM2G070038_P01           -TSLHEDSMHRLGHIP--------GRPRVDEGQ--AVQPALAGFPANQAAGLQVQSSLKPDIMATLAKLLPSVQSSPLVS           713
Sobic.002G264200.1.p        -ASLHEDSMQRLGHIP--------GRPRVDEGQ--AVQPALAGFPANQATGLQVQSSLKPDIMATLAKLLPSVQSSQLVS           685
AT2G43410.2                 -------PANASHSLYPPRENYIRGAPEHLTAA--SKPSVSEPLRIPNNAAPQAGVSLTPELLATLASILPATSQPAAPE           661
Glyma.11G126400.1.p         -RPLHEDSKLPAKPVYPPTGG-------PPPVH--SGPPD-YA-PNNTVAGSQAGVALTPELIATLASFLPTTTQSPATD           749
Solyc06g053320.2.1          -QVTLEDMKLPSKDYGSLTAAYATNTVQPSNSA--AYPSS-YVHQSNAAAPAQAGVSLTPELIANLVKILPASQLLSV-E           751
413458                      NTILPVCNMVQE-----KTVGAQDLLEREATIFQRSPDNRLYML------------PVTPQV-CEICRIAAASQYSQGAE           707
Pp3c7_3950V3.1.p            QHSLPHDGQP-AQTGKPTSVGLSDGLPSSVSNP--NSNSTSMLTQSQLANIAGLPGVLTPELIASLTALLPKTNMSQVSS           850
                                                                         : *:: . :  :

LOC_Os09g34070.1            GQLPLS---STD--QPALT----------------------------QMNDASTLAKVWRPENQAMASTS----------           721
Si028870m                   GQIPVN---STD--RP--S----------------------------QMHGLSMLSKVWNPENQSTTSNL----------           712
Pavir.J04458.1.p            GQMPMN---STD--RQ--S----------------------------QMHDPSMLSKAWNPENQSSASNL----------           713
GRMZM2G070038_P01           GQM--N---AID--RT--S----------------------------QIQDPSMLSKVWNPENQVTASNS----------           746
Sobic.002G264200.1.p        GQM--N---AIE--RP--S----------------------------QMQDPSMLSKVWTPENQVTASNS----------           718
AT2G43410.2                 SHQPMS---GPSTVVSTAHQS-------------------------NGLYNGEAPSQAWKRGPQTVHDASNQ--------           705
Glyma.11G126400.1.p         GAKSAV---GSSTMKPPFP---------------------------PMTPNDGNQSHLWKQDNQIADQSTHP--------           791
Solyc06g053320.2.1          GTTMPA---GASAGMPASD---------------------------VAVGPGKVQQQSWRYDQQAPGQAADH--------           793
413458                      AQDAGM---------IAIV-----------------------------------------------------EALRFCLKP           726
Pp3c7_3950V3.1.p            SADSNLVPPLVSKGSKTFTSSPGLPVGGAPALGGTHQNDVRPVFRPSQNNPPTEPAQGWQQQQNQQDRSQIGVSNFHLQA           930
                           .

LOC_Os09g34070.1            ----------SLEQIGNFQ-HSGQQFSKQAGAVHL----PNYGNLAGAQEHPTQH------SA--YNPEM-----TLNLP           773
Si028870m                   ----------SFGQIANLQ-QPGQQFSRQVSAAHL----TNYGSMVGAQEHSTQH------TA--YNPEV-----ALNLP           764
Pavir.J04458.1.p            ----------SFGQIANLQ-QPGQQFSRQASATHL----TNYGNMVGAPEHSTQH------TA--YNPEV-----ALNLP           765
GRMZM2G070038_P01           ----------SFGQMANVQ-HPGQQFSGQASAAHL----TNYGNMVSAQERSIQH------TA--YNPEV-----TLNLP           798
Sobic.002G264200.1.p        ----------SFGQIANVQ-HPGQQFSKQASAAHL----TNYGNMVSAQERSIQH------TA--YNPEV-----ALNLP           770
AT2G43410.2                 ----------SFQQY-------GNQYTP---AGQLPPPPSRYPPASNNPNYTSGMVH---GNM--QYQSQ-----SVNMP           755
Glyma.11G126400.1.p         -----------PQQL-------RSMYNIHNAHYQPYPPAS---APSGNPSQVVS------GSS--HIQDT-----AASMQ           837
Solyc06g053320.2.1          -------------MF-------SSQFNNQT---QVLPQLQAHPQVLNTPSHYSQGAT---SFN--QIQDH-----NLNLQ           840
413458                      CS---------------------------------SPASFVRSLSGQPS*-----------------------------           742
```

```
Pp3c7_3950V3.1.p          ADQNSMYTSQPPQQPHNLQPNPSQQLQLQQALQQ---PPHQNTQLQGQPSHMAGPPLSGQGSSFPGPPTHPQGYSAPGVQ         1007
                                                                        .


LOC_Os09g34070.1          PPPPP--PTL-PPSSAILSSQVGHS-LPTQ-MSQQQYQ-----------PE----QYYMTQSNYGQLATVSSSNLQAH--         831
Si028870m                 PPPP--IPTPTHSSTTTLPSQGGHS-LPTQ-TNQQL-------------PE----QYYVPQSNYVPLAMGSHSNIQAS--         821
Pavir.J04458.1.p          PPPPPPIPTPTHS-SITLPSQGGHS-LPTQ-LNQQLYQ-----------PE----QYYVPQSNYGPLATGSHSNLQAS--         825
GRMZM2G070038_P01         PPPPL--PTIPH-SSATLQSQGGHS-LPSQ-TNQQLYQ-----------PE----QYYVPQNNYGPLVPVSHSNLQIS--         856
Sobic.002G264200.1.p      PPPPL--PTEPH-RSATLPSQGGHS-LPTQ-INHQLYQ-----------PE----HYYVPQSNYGPLAPASHSNLQIS--         828
AT2G43410.2               QLSPLP--NMPH-NNYSMYTQGSSNHPVSQ-PMVQQYQ-----------PE----ASMPNQNY-GPI-------------         802
Glyma.11G126400.1.p       QQGAVS--SRHM-PNFMMPTQSG-QVAVSP-HASQNYQ-----------VE----VSPSNQKGFGVVQGTDASVLYNS--         895
Solyc06g053320.2.1        AQGGPP--QT-L-P----STINS-QVKVGK-DQTIIER-----------IS----LSRDDEDRDRVV*------------         882
413458                    --------------------------------------------------------------------------------         742
Pp3c7_3950V3.1.p          QFP--PLPQMPRPPQQPMGGPQQHMGAPPQLPSDQLAQLTALLTQRHQQPSQQQAASQLLQQHFQQNTTSQSSGLATAVS         1085


LOC_Os09g34070.1          -------HQQIVATPAAQA----PVA--------AQFP------PAMQ------------------APA-----------         857
Si028870m                 -------NANNPAPPLPQVYPGPPANNQ--MGNLPQLQ------P-------------SSH-----GQQ-----------         857
Pavir.J04458.1.p          -------NANNLAPPLRQVNPGPPANNQ--MGNLPQLQ------P-------------SSH-----GQQ-----------         861
GRMZM2G070038_P01         -------NTNNPTLTIPQVNPGPPTNNQ--IGNLAQPQ------HSMPLHVDRASQDFSSQ-----GQQ-----------         905
Sobic.002G264200.1.p      -------NTNNPTPAIPQVNPGPPT-NQ--IGNLAQLQ------HSMPLHVDRASQDFSSQ-----LQQ-----------         876
AT2G43410.2               -------------PSYQQANFHGVTTNQAQNLNPSQFQ------AAMQPPADKANLEPQNQ-----ALR-----------         847
Glyma.11G126400.1.p       ---QAFQQPNNNSLAFQQPNNSFALSNQVNSTNASQQQ------TAMPYTVDQVNPDTPNQ-----Q-------------         948
Solyc06g053320.2.1        --------------------------------------------------------------------------------         882
413458                    --------------------------------------------------------------------------------         742
Pp3c7_3950V3.1.p          QPQQGLGPQSQSQQQQHQPPLG-PPSQQPQPPNYSQYSQSPWGQGSAPPSSGHGNSQLSSLVPQLQQQQNSSNVQSGSWE         1164


LOC_Os09g34070.1          -------A--AQAPVAAQA-SADEAERNRKYQATLQLAQRLLGQLQQKP-GNQP*--------      900
Si028870m                 -------Q--HFAPG-TAQ-APDEADKSKKYQATLQLAQNLLLQIQQRQSGNQP*--------      900
Pavir.J04458.1.p          -------Q--HFAPG-TAQ-APDEADKSKKYQATLQLAQNLLLQIQR-QSGNQS*--------      903
GRMZM2G070038_P01         -------Q--NRGPG-AAQ-APE-EDKSKKYQATLQLAQNLLLQLQQRGSGNQS*--------      947
Sobic.002G264200.1.p      -------Q--NLGPG-AAQ-APE-EDKSKKYQATLQLAQNLLLQIQQRGSGNQS*--------      918
AT2G43410.2               -------LQPMISGDGQGT-TDGEVDKNQRYQSTLQFAANLLLQIQQKQQQQSSGTPAGQGP*      901
Glyma.11G126400.1.p       ---------LPMFGVSQGQ-TEVEADKNQRYQSTLQFAANLLLQIQQQQQQAPGGHGPGQ*--      998
Solyc06g053320.2.1        ---------------------------------------------------------------      882
413458                    ---------------------------------------------------------------      742
Pp3c7_3950V3.1.p          GSLSQASAGAELQASSQNQGGSEADAQTKRFQATVQLAAALLQQMQQQQGKPSGGQEQR*---      1223
```

LOC_Os12g02310::732978-734220 Os12g0115000 "LTPL11 - Protease inhibitor/seed storage/LTP family protein precursor, expressed"

```
LOC_Os12g02310.1        -----------------------------------------------------MARAQLVLV--ALVAALLLAGPHTTMA    25
Si011376m               -----------------------------------------------------MARAQV-V--VLAVVAVVLLAAAASEA    24
Pavir.Cb02192.1.p       MDKEWPPIIVSSSRPSSGLGYLKTNIPLHTLLTSACNNSSIQHRIVPSSHSRTMARAQQVVAIAVVAAVVLLAAAATSEA    80
GRMZM2G101958_P01       --------------------------------------------------MARMQKLAV-ATAAVV-ALVLLAAAATSEA    28
Sobic.008G030900.1.p    -----------------------------------------------------MARLAV-AIAVVAAAAVVVLAATTSEA    26
AT2G38540.1             -----------------------------------------------------MA--GVMKLACLLLACMIVAGPITSNA    25
Glyma.03G040400.1.p     ----------------------------------------------------MMTSFKVACVVAMMCMVVAMSVTPMAQA    28
Solyc10g075070.1.1      -----------------------------------------------------MEMSSKIA----CFIVLCMIVVAPHGE    23
427417                  -----------------------------------------------------MGGARILVVV--ALLASLLLHVGLVAA    25
                                                                  *              :

LOC_Os12g02310.1        AISCGQVNSAVSPCLSYARG-GS-GPSAACCSGVRSLNSAATTTADRRTACNCLKNVAGSI-SGLNAGNAASIPSKCGVS    102
Si011376m               AISCGQVNSAIGQCLPYARGQGS-KPSDACCSGVKRLNSAATTTADRRAACNCLKNAARGI-SGLNAGNAASIPSKCGVS    102
Pavir.Cb02192.1.p       AVTCGQVNSAIGPCIAYARGSGS-GPSSACCSGVRSLNSAARSTADRRAACNCLKSAAGRV-SGLNAGNAASIPSKCGVS    158
GRMZM2G101958_P01       AISCGQVASAIAPCISYARGQGS-GPSAGCCSGVKSLNNAARTTADRRAACNCLKNAAAGV-SGLNAGNAASIPSKCGVS    106
Sobic.008G030900.1.p    AISCGQVSSAIAPCLSYARGQGS-APSAGCCSGVRSLNSAARTTADRRAACNCLKNAARGI-SGLNAGNAASIPSKCGVS    104
AT2G38540.1             ALSCGSVNSNLAACIGYVLQ-GG-VIPPACCSGVKNLNSIAKTTPDRQQACNCIQGAARALGSGLNAGRAAGIPKACGVN    103
Glyma.03G040400.1.p     AITCGQVAGDVSPCLSYLRS-GG-KPSDACCNGVKSLSGAAKTTADRQAACNCLKNLANNMGQSLNAGNAASLPGKCGVN    106
Solyc10g075070.1.1      ALSCGQVESGLAPCLPYLQG--K-GPLGGCCRGVKGLLGAAKTPADRKTACTCLKSAANAI-KGLNLGKAAGIPSACGVS    99
427417                  AVDCSAAQQAMFPCLSAAVGGNPPPPSVACCAAMKSVS--------KLEMCQCLVNQTSTV-PGLNMTAARNIPANCNIS    96
                        *: *. .  :  *:           .** .:: :        :  * *:  :  :  .**   *  :*  * :.

LOC_Os12g02310.1        IPYTISPSIDCSSVN*-    117
Si011376m               IPYSISTSTDCSRVS*-    117
Pavir.Cb02192.1.p       IPYTISPSVDCSKVA*-    173
GRMZM2G101958_P01       IPYTISTSTDCSRVN*-    121
Sobic.008G030900.1.p    VPYTISTSTDCSRVN*-    119
AT2G38540.1             IPYKISTSTNCKTVR*-    118
Glyma.03G040400.1.p     IPYKISTSTNCATIKF*    122
Solyc10g075070.1.1      IPYKISPFTDCSKVQ*-    114
427417                  AA------ADCS*----    102
                              :*
```
