# Supplementary material for: Low mutation rate of spontaneous mutants enables detection of causative genes by comparing whole genome sequences
Source: Front Plant Sci. 2024 Apr 4;15:1366413. doi: 10.3389/fpls.2024.1366413 (PMC11024370; doi:10.3389/fpls.2024.1366413)
Supplement: Supplementary file 7 [file Table_1.docx]

Supplemental Table 1 List of DNA polymorphisms found between Ginbouzu and three related varieties

| CHROM | POS | REF | ALT | Position within the candidate gene | Impact | RAP | RGAP | HGVS.p | Evaluation of genes |
| --- | --- | --- | --- | --- | --- | --- | --- | --- | --- |
| chr02 | 24351783 | G | A | missense_variant | MODERATE | Os02g0615800 | LOC_Os02g40240 | p.Ser244Asn | Annotated as receptor kinase-like protein Xa21. These amino acid substitutions would not be important because none of amino acid residues are conserved in other plant species.  In addition, as this gene is specifically expressed in leaf blades, it is difficult to imagine it impacting on culm length. |
| chr02 | 24351797 | C | A | missense_variant | MODERATE | Os02g0615800 | LOC_Os02g40240 | p.Leu249Ile |  |
| chr02 | 24351798 | T | C | missense_variant | MODERATE | Os02g0615800 | LOC_Os02g40240 | p.Leu249Pro |  |
| chr02 | 24351813 | A | T | missense_variant | MODERATE | Os02g0615800 | LOC_Os02g40240 | p.Tyr254Phe |  |
| chr02 | 24351830 | G | A | missense_variant | MODERATE | Os02g0615800 | LOC_Os02g40240 | p.Val260Ile |  |
| chr02 | 24351831 | T | C | missense_variant | MODERATE | Os02g0615800 | LOC_Os02g40240 | p.Val260Ala |  |
| chr02 | 24351873 | T | C | missense_variant | MODERATE | Os02g0615800 | LOC_Os02g40240 | p.Ile274Thr |  |
| chr02 | 24351879 | G | A | missense_variant | MODERATE | Os02g0615800 | LOC_Os02g40240 | p.Arg276His |  |
| chr06 | 22075723 | T | A | missense_variant | MODERATE | Os06g0570100 | LOC_Os06g37364 | p.Arg238Ser | Annotated as ent-kaurene oxidase-like  2. This is the causal gene of short culm. Alighnment is shown in Supplemental Figure 3. |
| chr06 | 28860394 | G | GTCGT CGA | frameshift_variant | HIGH | Os06g0691900 |  | p.Thr62fs | Annotated as similar to F-box domain containing protein. It is highly  expressed in root (TENOR),and pollen (Rice X pro). |
| chr06 | 30092923 | G | A | missense_variant | MODERATE | Os06g0711700 | LOC_Os06g49750 | p.Ala186Val | The polymorphism is observed not only in Ginbozu-Miide but also in all three  other lines belonging to the Ginbozu CMPLX. Therefore, we cannot assume that this polymorphism is the cause of the short culm. |
| chr07 | 2454978 | G | A | missense_variant | MODERATE | Os07g0147500 | LOC_Os07g05360 | p.Gly88Glu | Annotated as similar to Photosystem II 10 kDa polypeptide. The  overexpression of this gene did not change the culm length. |
| chr07 | 24470890 | A | G | missense_variant | MODERATE | Os07g0599500 | LOC_Os07g40850 | p.Val51Ala | Annotated as 'hypothetical' in both  RAP and MSU, but no homologs have been found in other plants. |
| chr09 | 5105182 | GATTT ATCTT | G | splice_acceptor_v ariant&splice_regio n_variant&5_prime  _UTR_variant&intro n_variant | HIGH | Os09g0267701 |  |  | Annotated as hypothetical protein in RAP, and no annotation in MSU. |
| chr11 | 23333817 | CGGG  GATCA | C | frameshift_variant  &start_lost | HIGH | Os11g0605100 | LOC_Os11g39190 | p.Met1fs | Annotated as NB-ARC domain containing protein. It is highly  expressed in leaf blade but not highly expressed stem and leaf sheath, almost not expressed in inflorescence in Rice X pro. |
| chr11 | 23333874 | A | C | missense_variant | MODERATE | Os11g0605100 | LOC_Os11g39190 | p.Glu17Ala |  |
| chr11 | 23333876 | G | T | missense_variant | MODERATE | Os11g0605100 | LOC_Os11g39190 | p.Ala18Ser |  |
| chr11 | 23333877 | C | T | missense_variant | MODERATE | Os11g0605100 | LOC_Os11g39190 | p.Ala18Val |  |
| chr11 | 28812304 | T | TC | frameshift_variant | HIGH | Os11g0704100 | LOC_Os11g47780 | p.Lys1046fs | Annotated as NB-ARC domain containing protein. None of the amino acid residues conserved in other plant species. |
| chr11 | 28812421 | A | T | missense_variant | MODERATE | Os11g0704100 | LOC_Os11g47780 | p.Val1007Glu |  |
| chr11 | 28812422 | C | G | missense_variant | MODERATE | Os11g0704100 | LOC_Os11g47780 | p.Val1007Leu |  |
| chr11 | 28812430 | A | C | missense_variant | MODERATE | Os11g0704100 | LOC_Os11g47780 | p.Met1004Arg |  |
