# Supplementary material for: Low mutation rate of spontaneous mutants enables detection of causative genes by comparing whole genome sequences
Source: Front Plant Sci. 2024 Apr 4;15:1366413. doi: 10.3389/fpls.2024.1366413 (PMC11024370; doi:10.3389/fpls.2024.1366413)
Supplement: Supplementary file 8 [file Table_2.docx]

Supplemental Table 2 List of DNA polymorphisms found between Kamenoo-Daikoku and five related varieties.

| CHROM | POS | REF | ALT | Position within the  candidate gene | Impact | RAP | RGAP | HGVS.p | Evaluation of polymorphisms |
| --- | --- | --- | --- | --- | --- | --- | --- | --- | --- |
| chr01 | 3070183 | T | G | missense_variant | MODERATE | Os01g0158600 | LOC_Os01g06520 | p.Lys318Asn | Annotated as Leucine-rich repeat, N-terminal domain containing. No polymorphisms are found in Kamenoo CMPLX. |
| chr01 | 3070194 | T | G | missense_variant | MODERATE | Os01g0158600 | LOC_Os01g06520 | p.Lys315Gln |  |
| chr01 | 3070213 | A | C | missense_variant | MODERATE | Os01g0158600 | LOC_Os01g06520 | p.Phe308Leu |  |
| chr01 | 3070214 | A | C | missense_variant | MODERATE | Os01g0158600 | LOC_Os01g06520 | p.Phe308Cys |  |
| chr01 | 3070224 | GGT | G | frameshift_variant | HIGH | Os01g0158600 | LOC_Os01g06520 | p.Asp304fs |  |
| chr01 | 3070227 | C | CCA | frameshift_variant | HIGH | Os01g0158600 | LOC_Os01g06520 | p.Asp304fs |  |
| chr01 | 3070233 | G | A | missense_variant | MODERATE | Os01g0158600 | LOC_Os01g06520 | p.Arg302Cys |  |
| chr01 | 3070263 | A | T | missense_variant | MODERATE | Os01g0158600 | LOC_Os01g06520 | p.Phe292Ile |  |
| chr01 | 3070283 | A | G | missense_variant | MODERATE | Os01g0158600 | LOC_Os01g06520 | p.Leu285Pro |  |
| chr01 | 42217563 | C | T | missense_variant | MODERATE | Os01g0957900 | LOC_Os01g72780 | p.Pro172Leu | Annotated as Armadillo-type fold domain containing. The Pro residue was not conserved in other plant species. |
| chr05 | 15612964 | T | A | missense_variant&s plice_region_variant | MODERATE | Os05g0333200 | LOC_Os05g26890 | p.Gln58Leu | Annotated as α-subunit of GTP- binding protein. This is the causal gene of short brown rice length. Alignment is shown in Supplemental Figure 5. |
| chr06 | 1047315 | GC | G | frameshift_variant | HIGH | Os06g0119300 | LOC_Os06g02850 | p.Ala429fs | Annotated as protein of unknown function DUF594 family. This gene  (Os06t0119300/LOC_Os06g028  50) has two mutations  (chr06:1047315:GC to G,  chr06:1047331:A to G) only in Kamenoo-Daikoku. However, when we looked at homologs and orthologs of this gene in other plants, only O. sativa  (both japonica and indica) has a duplication in this gene. This gene can be removed from the list of candidates as it is functionally redundant. |
| chr06 | 1047331 | A | G | missense_variant | MODERATE | Os06g0119300 | LOC_Os06g02850 | p.Phe424Leu |  |
| chr06 | 1874358 | G | C | missense_variant | MODERATE | Os06g0134800 | LOC_Os06g04380 | p.Pro230Arg | Annotated as folate-binding, YgfZ domain containing. This proline is well conserved among plant species. However, it is unlikely that it affects seed size. The gene is highly  expressed in the shoot under dry conditions, but its  expression in other organs is low (TENOR), and its  expression in inflorescence is not pronounced (Gramene). |
| chr07 | 26806691 | A | G | missense_variant | MODERATE | Os07g0644000 | LOC_Os07g44940 | p.Ser309Pro | Annotated as ATP12, ATPase F1F0-assembly protein. This serine is not conserved in  Nicotiana. It is not an important amino acid. |
| chr09 | 11392980 | C | G | missense_variant&s plice_region_variant | MODERATE | Os09g0355400 | LOC_Os09g18594 | p.Ala548Gly | Annotated as Malectin domain protein kinase, Resistance to sheath blight disease. This alanine is not conserved in  other plant species. |
| chr11 | 27796136 | C | T | missense_variant | MODERATE | Os11g0686500 | LOC_Os11g45930 | p.Leu685Phe | Annotated as NB-ARC domain containing. This leucine is well conserved, however, this gene is highly expressed in root (TENOR), root and leaf blade (Rice x Pro). |
