# Supplementary material for: Low mutation rate of spontaneous mutants enables detection of causative genes by comparing whole genome sequences
Source: Front Plant Sci. 2024 Apr 4;15:1366413. doi: 10.3389/fpls.2024.1366413 (PMC11024370; doi:10.3389/fpls.2024.1366413)
Supplement: Supplementary file 9 [file Table_3.docx]

Supplemental Table 3 List of DNA polymorphisms found between two awnless (Mubo-Aikoku and Tokyo-Mubo-Aikoku) and five awned varieties.

| CHROM | POS | REF | ALT | Position within the  candidate gene | Impact | RAP | RGAP | HGVS.p | Evaluation of polymorphisms |
| --- | --- | --- | --- | --- | --- | --- | --- | --- | --- |
| 6 | 29079888 | G | GGCCGAGGCT AGAGGTGGCC AAACGGGCCG GGCCGCCCGT TTGGCCACCTC TA,GGCCGAG GCTAGAGGTG GCCAAACGGG CCGTGCCGGA CCCGTGCCCG TGCCGTGCCG GGCCGGGCC GCCCGTTTGG CCACCTCTA | frameshift_variant | HIGH | Os06g0695900 | LOC_Os06g48065 | p.Arg124fs | Annotated as Zinc finger, RING-type, C3HC4 type. More details are given below. |
| 8 | 11267910 | CAATCT CAACA GTCAA  TCACAT | C | frameshift_variant | HIGH | Os08g0285350 | LOC_Os08g18890 | p.Ile57fs | Annotated as 'hypothetical' in both RAP and MSU, but no homologs have been found in other plants. |
| 10 | 2554010 | C | CTCCTACAG | frameshift_variant | HIGH | Os10g0141700 | LOC_Os10g05210 | p.Leu13fs | There is no gene encoding a protein similar to that predicted by RAP  (Os10t0141700-01), while MUS (LOC_Os10g05210.2) predicts a different region encoding an F-box domain-containing protein with high similarity to other plants in the grass family. The polymorphism is in the 5' UTR of the MUS prediction. |
| 6 | 6027928 | G | C | missense_variant | MODERATE | Os06g0218000 | LOC_Os06g11420 | p.Gly36Arg | Annotated as Ski-interacting protein, but RAP predicts this as most likely nonfunctional or a pseudogene. |
| 6 | 15337074 | G | T | missense_variant | MODERATE | Os06g0367100 | LOC_Os06g26234 | p.Met130Ile | Annotated as glycoside hydrolase/1,4-alpha-glucan-branching enzyme similar to starch branching enzyme III by RAP. M130I is also found in its homolog in Prunus persica. Alignment is shown in Supplemental Dataset. |
| 8 | 27025886 | G | T | missense_variant | MODERATE | Os08g0539900 | LOC_Os08g42710 | p.Val530Phe | Annotated as NB-ARC domain containing by RAP and MSU, but no homologues have been found in other plants. |
| 8 | 27025889 | C | T | missense_variant | MODERATE | Os08g0539900 | LOC_Os08g42710 | p.Leu531Phe | Annotated as NB-ARC domain containing by RAP and MSU, but no homologues have been found in other plants. |
| 12 | 733469 | C | G | missense_variant | MODERATE | Os12g0115000 | LOC_Os12g02310 | p.Thr65Ser | Annotated as Lipid transfer protein Class I/Lipid transfer protein b1 by RAP, and LTPL11 - Protease inhibitor/seed storage/LTP family protein precursor by MSU. T65S is also found in its homolog of various plants, such as Zm00008a014788, Pavir.3KG042100.1, Sobic.008G031000.1,  HORVU5Hr1G046520.1, ect. Alignment is shown in Supplemental Dataset. |
| 12 | 6643624 | A | G | missense_variant | MODERATE | Os12g0222650 | LOC_Os12g12115 | p.Met60Thr | Annotated as hypothetical by RAP, and expressed protein by MSU. no homologues have been found in other plants. |
