# Supplementary material for: Low mutation rate of spontaneous mutants enables detection of causative genes by comparing whole genome sequences
Source: Front Plant Sci. 2024 Apr 4;15:1366413. doi: 10.3389/fpls.2024.1366413 (PMC11024370; doi:10.3389/fpls.2024.1366413)
Supplement: Supplementary file 10 [file Table_4.docx]

Supplemental Table 4 List of DNA polymorphisms found between awnless Bozu-Aikoku and five awned varieties.

| CHROM | POS | REF | ALT | Position within the candidate gene | Impact | RAP | RGAP | HGVS.p | Evaluation of polymorphisms |
| --- | --- | --- | --- | --- | --- | --- | --- | --- | --- |
| 3 | 885141 | C | CGGCA | frameshift_variant | HIGH | Os03g011570 0 | LOC_Os03g02460 | p.Ser299fs | Annotated as Short-chain dehydrogenase/reductase SDR family by RAP, and retinol dehydrogenase by MSU. More details are given below. |
| 12 | 5857228 | T | TGCAG | frameshift_variant | HIGH | Os12g021150 0 | LOC_Os12g10870 | p.Glu679fs | Annotated as LRR-N-terminal domain by RAP, and verticillium wilt disease resistance by MSU. There is a report that this gene is upregulated by agricultural chemicals  (https://shingi.jst.go.jp/pdf/2014/okayama05.pdf). |
| 3 | 21818130 | T | C | missense_variant | MODERATE | Os03g058950 0 | LOC_Os03g39270 | p.Ile10Val | Annotated as Conserved hypothetical/expressed, but no homologs have been found in other plants. |
| 4 | 32297942 | C | T | missense_variant | MODERATE | Os04g063500 0 | LOC_Os04g54230 | p.Ala29Thr | Annotated as similar to H0315F07.10 by RAP, and wound induced by MSU. Alignment is shown in Supplemental Dataset. |
| 5 | 2967670 | C | T | missense_variant | MODERATE | Os05g015115 0 | LOC_Os05g05920 | p.Arg308Gln | Annotated as hypothetical by RAP (but this prediction could be incorrect because of no homologs in other plants), and  desiccation-related protein PCC13-62 precursor by MSU. In MSU prediction, this SNP causes S32L located in nonconserved region. Alignment is shown in Supplemental Dataset. |
| 9 | 20110707 | G | A | missense_variant &splice_region_va riant | MODERATE | Os09g051630 0 | LOC_Os09g34070 | p.Ala830Val | Annotated as Spen (Split ends)-like protein, Vegetative to reproductive transition by RAP, and RNA recognition motif containing protein by MSU. A830 is not conserved among grasses. Alignment is shown in Supplemental Dataset. |
| 10 | 22591837 | C | T | missense_variant | MODERATE | Os10g056980 0 | LOC_Os10g42020 | p.Arg85Cys | Annotated as Similar to RIR1b protein by RAP, and RALFL29 - Rapid ALkalinization Factor RALF family protein precursor by MSU. No homologs have been found in other plants. |
| 12 | 10050436 | A | G | missense_variant | MODERATE | Os12g027370 0 | LOC_Os12g17530 | p.Thr303Ala | Annotated as Conserved hypothetical protein, and expressed protein by MSU. T303A also occurs in its homolog,  Seita.3G306400.1. |
